# *EFTUD2* gene deficiency disrupts osteoblast maturation and inhibits chondrocyte differentiation via activation of the p53 signaling pathway

**DOI:** 10.1186/s40246-019-0238-y

**Published:** 2019-12-05

**Authors:** Jing Wu, Yi Yang, You He, Qiang Li, Xu Wang, Chengjun Sun, Lishun Wang, Yu An, Feihong Luo

**Affiliations:** 1 Department of Pediatric Endocrinology and Inherited Metabolic Diseases, Children’s Hospital of Fudan University, Shanghai, 201102 China; 2 Institute of Pediatrics, Children’s Hospital of Fudan University, Shanghai, 201102 China; 3 Shanghai Synchrotron Radiation Facility, Shanghai Institute of Applied Physics, Chinese Academy of Sciences, 239 Zhangheng Road, Pudong District, Shanghai, 201204 China; 4 Translational Medical Center for Development and Disease, Shanghai Key Laboratory of Birth Defect, Institute of Pediatrics, Children’s Hospital of Fudan University, Shanghai, 201102 China; 5 Key Laboratory of Metabolism and Molecular Medicine, Ministry of Education, and Department of Biochemistry and Molecular Biology, School of Basic Medical Sciences, Fudan University, Shanghai, 200032 China; 6 Institute of Fudan-Minhang Academic Health System, Minhang Hospital, Fudan University, 170 Xinsong Road, Shanghai, 201199 China; 7 Human Phenome Institute, Fudan University, 825 Zhangheng Road, Shanghai, 201203 China

**Keywords:** EFTUD2, Mandibulofacial dysostosis with microcephaly, Developmental delay, *TP53* signaling pathway, Zebrafish, Osteoblast, Chondrocyte

## Abstract

**Background:**

Mandibulofacial dysostosis with microcephaly (MFDM) is characteristic of multiple skeletal anomalies comprising craniofacial anomalies/dysplasia, microcephaly, dysplastic ears, choanal atresia, and short stature. Heterozygous loss of function variants of *EFTUD2* was previously reported in MFDM; however, the mechanism underlying *EFTUD2*-associated skeletal dysplasia remains unclear.

**Results:**

We identified a novel frameshift variant of *EFTUD2* (c.1030_1031delTG, p.Trp344fs*2) in an MFDM Chinese patient with craniofacial dysmorphism including ear canal structures and microcephaly, mild intellectual disability, and developmental delay. We generated a zebrafish model of *eftud2* deficiency, and a consistent phenotype consisting of mandibular bone dysplasia and otolith loss was observed. We also showed that *EFTUD2* deficiency significantly inhibited proliferation, differentiation, and maturation in human calvarial osteoblast (HCO) and human articular chondrocyte (HC-a) cells. RNA-Seq analysis uncovered activated *TP53* signaling with increased phosphorylation of the TP53 protein and upregulation of five *TP53* downstream target genes (*FAS*, *STEAP3*, *CASP3*, *P21*, and *SESN1*) both in HCO and in *eftud2*−/− zebrafish. Additionally, inhibition of *p53* by morpholino significantly reduced the mortality of *eftud2*−/− larvae.

**Conclusions:**

Our results confirm a novel de novo variant of the *EFTUD2* gene and suggest that *EFTUD2* may participate in the maturation and differentiation of osteoblasts and chondrocytes, possibly via activation of the TP53 signaling pathway. Thus, mutations in this gene may lead to skeletal anomalies in vertebrates.

## Background

Mandibulofacial dysostosis with microcephaly (MFDM, MIM# 610536) is a rare syndrome with a wide spectrum of congenital anomalies [1–3]. The main clinical features are characteristic facial features and associated craniofacial malformations comprising malar and mandibular hypoplasia, micrognathia, dysplastic ears, cleft palate, choanal atresia, and microcephaly, which are consequences of skeletal development impairment of the head and face [1–6]. Other extracranial abnormalities, including choanaloresophageal atresia, congenital heart disease, limb defects, intellectual disability, and short stature, are frequently presented in MFDM [1, 7, 8]. As a result of skeletal dysplasia, some patients may present respiratory difficulty, hearing loss, and developmental delays [5, 6, 9, 10].

The heterozygous pathogenic variant or deletion in the elongation factor Tu GTP-binding domain-containing 2 gene (*EFTUD2*, MIM# 603892) is considered the cause of MFDM [9–12]. Thus far, 95 *EFTUD2* variants have been reported previously in MFDM patients, and most of them are de novo [11]. An animal model of *eftud2* deficiency in vertebrates could mimic some clinical phenotypes of abnormal morphology, such as small head/eye and curved bodies [13]. However, the mechanism by which haploinsufficiency of *EFTUD2* leads to bone deformities has yet to be explored.

In this study, we identified a novel de novo *EFTUD2* null variant in a Chinese patient by whole-exome sequencing (WES) analysis. We generated a zebrafish line with *eftud2* disruption to investigate the facial morphology, which presents mandibulofacial anomalies in patients, and more importantly, to determine the molecular mechanism of bone dysplasia.

## Materials and methods

The details of DNA sequencing, microinjection of zebrafish embryos, TALEN efficiency testing in vitro, DNA extraction from zebrafish, real-time PCR, and Western blotting are described in the supplementary methods.

### Subjects and DNA extraction

A 3-year-old boy and his family were enrolled in this study. Written informed consents were obtained from his family, and this study was approved by the ethics committee of the Children’s Hospital of Fudan University in Shanghai, China. A direct physical exam, computerized tomography scan (CT), and the Wechsler Intelligence Scale for Children (WISC) were performed on the proband to verify the phenotypic characteristics of the disorder. Genomic DNA of the proband and his parents was isolated from whole blood using a Qiagen mini blood kit (Qiagen, Germany).

### Mutation analysis

After whole-genome array-CGH (a-CGH) analysis and WES, variant calling was performed using NextGENe® (SoftGenetics, PA, USA) with default parameters based on the FASTQ format files generated from Illumina software. Each read was aligned to the reference genome (Human 37.3, SNP135). Single nucleotide variants (SNVs) and indels were identified and saved in VCF format. These SNVs and indels were used for further interpretation analysis using Ingenuity® Variant Analysis™ (Ingenuity Systems, CA, USA). The filter cascade was performed by common variants with MAF > 0.01 (1000 Genomes and NHLBI GO Exome Sequencing Project), genetic analysis (dominant and recessive variants), predicted deleterious (SIFT and Polyphen2), variant annotation (OMIM, COSMIC, and TCGA), and biological context. Protein structures were modeled through the Zhang Lab online system (http://zhanglab.ccmb.med.umich.edu/).

### Morpholino design and *EFTUD2* gene mRNA synthesis

An *eftud2* morpholino (EMO) targeting a splicing site of the gene and a corresponding mismatch control morpholino (EMIS-MO) were designed (www.gene-tools.com) and synthesized: EMO 5′ CAGGTTATACAATCACCTCATCAGC 3′; EMIS-MO 5′ CAGaTTATAaAATaACCTaATCAaC 3′. To identify the activation of the p53 pathway in *eftud2* knockout zebrafish, a morpholino oligonucleotide corresponding to P53 (5′ GCGCCATTGCTTTGCAAGAATTG 3′) was synthesized as previously described [14]. For mRNA preparation, 100 μg/μl *eftud2* mRNA was obtained from the linearized PCS2+ plasmid template carrying the full-length human normal *EFTUD2* cDNA, using the mMESSAGEmMACHINE Kit (Ambion, USA). Details of the injection and validation of effectiveness tested by PCR amplification using EMO1 and EMO2 primers are shown in supplementary materials.

### TALEN mRNA design and generation

Three transcription activator-like effector nucleases (TALENs) were designed to disrupt the first two exons of the *eftud2* gene in zebrafish with the following sequences: *eftud2*-T1: 5′-TGATCTTTATGACGAGTTtggaaattatatcggACCAGAGCTGGACTCTG-3′; *eftud2*-T2: 5′-ATCGGACCAGAGCTGGActctgatgaggatgaAGAGCTAGATGCAGAGG-3′; and *eftud2*-T3: 5′-GGAGGTGGTTCTGCATGaggataaaaagtattatccCACTGCTGAAGAAGTGT-3′. TALEN efficiency first was tested in vitro by luciferase activity detection (seen in supplementary methods), and the final TALEN plasmids were constructed in the PCS2+ backbone. After the in vitro transcription of the TALENs using the mMESSAGE mMACHINE SP6 Transcription Kit (Ambion, USA), TALEN mRNAs were purified using phenol-chloroform methods, resuspended at a concentration of 100 μg/μl in nuclease-free H_2_O, and stored at − 80 °C before microinjection into zebrafish embryos (AB strain).

### shRNA construction and viral transfection

EFTUD2 knockdown was conducted by shRNA using the PLKO.1 purolentiviral vector. The sequences of the shRNAs targeting the *EFTUD2* gene were as follows: EFTUD2-sh1 5′-GCCTCTCACAGAACCCATTAT-3′; EFTUD2-sh2 5′-CCCATTATTAAGCCAGTGAAA-3′; and EFTUD2-sh3 5′-GCTTTGCTGAAACGCCTAATA-3′. A scrambled shRNA (shNT, 5′-GCTTTGCTGAAACGCCTAATA-3′) was employed as a control. Lentiviral particles were generated using a method employed in previous studies [15]. Cells were replated in 6-well plates and transfected at MOI = 3 in the presence of 8 μg/ml polybrene.

### Zebrafish breeding and cell cultivation

The maintenance and microinjection of zebrafish were carried out as described in a previous study [16]. Morpholinos or TALEN mRNAs were injected into one-cell stage embryos to obtain F0 mosaics. After further out-crossing of F0 mosaics with wild type (WT) to generate heterozygous *eftud2* mutant offspring (F1), we obtained the F2 generation from F1 interbreeding. This study was approved by the Ethics Committee of Children’s Hospital of Fudan University ([2014] No. 115).

The human calvarial osteoblast (HCO) and human articular chondrocyte (HC-a) primary cell lines were purchased from ScienCell Research Laboratories (ScienCell, USA). HCO cells were cultured in specific medium (ScienCell, USA) containing 5% FBS (0025, ScienCell, USA), 1% osteoblast growth supplement (4652, ScienCell, USA), and 1% penicillin/streptomycin (0503, ScienCell, USA), whereas HC-a cells were cultured in medium containing 5% FBS, 1% chondrocyte growth supplement (4682, ScienCell, USA), and 1% penicillin/streptomycin, at 37 °C in a humidified 5% CO_2_ atmosphere.

### Zebrafish and cellular alcian blue and alizarin red staining

#### Zebrafish staining

The cartilage and bone of zebrafish were double-stained with an acid-free solution containing alcian blue 8GX and alizarin red S at the same time according to a previously reported method [17] and imaged under a dissecting microscope (Zeiss, Germany).

#### Cell staining

HCO and HC-a cells were stained with alizarin red S and alcian blue 8GX solution separately at 3 days after cell confluence. Briefly, HCO cells plated in 6-well plates were washed in PBS (phosphate buffer saline) and fixed in 4% paraformaldehyde (PFA) for 15 min, washed again in PBS 3 times, stained with 0.5% alizarin red S for 1 h, and then rinsed with PBS. The procedure for HC-a staining was similar to that for HCO staining, except that staining with the alcian blue 8GX solution was conducted for 30 min, after which the samples were washed 3 times with 3% glacial acetic acid for 3 min each before being washed in PBS and photographed with an inverted microscope (Leica, Germany).

### Proliferation assay using Cell Titer-Glo

For this assay, 2 × 10^4^ cells per well were plated in a 96-well plate and transfected with lentiviruses for 5 h after cell adherence. The proliferation of HCO and HC-a cells was assessed indirectly at 3 days before cell confluence by detecting the amount of adenosine triphosphate (ATP) in viable cells according to the manufacturer’s instructions (Promega, USA). Then, 100 μl of the Cell Titer-Glo reagent was added to 100 μl of culture medium, and fluorescence was measured with a luminescence detection system (BioTek, USA).

### ALP activity assay

ALP enzyme activity was determined using the Alkaline Phosphatase Activity Fluorometric Assay Kit (BioVision, USA). Briefly, cells were plated in a 6-well plate at a density of 2 × 10^5^ cells per well. Cell lysates were subsequently prepared in 400 μl of assay buffer at 3 days before or 2 weeks after cell confluence. After cell lysates were centrifuged at 13,000*g* for 3 min, 110 μl of sample lysates was mixed with 20 μl of 0.5 mM 4-methylumbelliferyl phosphate disodium salt (MUP). A standard curve was produced based on a concentration series of 0, 0.1, 0.2, 0.3, 0.4, and 0.5 nM MUP. All reactions were incubated in the dark at room temperature and stopped with 20 μl of stop solution. The results were read at 360-nm excitation/460-nm emission (BioTek, USA).

### RNA sequencing analysis

Total RNA was isolated and purified from HCO cells transfected with sh2 and shNT 3 days before cell confluence was observed using the Qiagen RNeasy Mini Kit (QIAGEN, Germany), after which 7 μg of total RNA was used for library preparation. The total RNA was further purified with the RNeasy Micro Kit (QIAGEN, Germany) and RNase-Free DNase Set (QIAGEN, Germany) and sequenced on the Illumina HiSeq2500 platform. Hierarchical clustering analysis of DEGs (differentially expressed genes) was performed to explore gene expression patterns between the two groups. Differentially expressed genes were determined based on the following criteria: FDR ≤ 0.05, Fold-change ≥ 2, with the FPKM value of each gene calculated by Cufflinks (version: 2.1.1). To analyze and acquire functional information for DEGs, gene ontology (GO) and Kyoto Encyclopedia of Genes and Genomes pathway (KEGG) enrichment analyses were performed using R, based on the hypergeometric distribution. Additionally, information on protein-protein interactions was evaluated with the Search Tool for the Retrieval of Interacting Genes (STRING: http://string-db.org/).

### Synchrotron X-ray microtomography analysis

Synchrotron radiation X-ray microtomography (SR-μCT) was performed with the BL13W1 beam line in the Shanghai Synchrotron Radiation Facility (SSRF). Adult zebrafish were anaesthetized and fixed in 4% PFA for a week, followed by dehydration in a series of ethanol dilutions ranging from 75 to 100% ethanol. The specimens were stored vertically in plastic tubes, which were mounted on a sample stage and imaged with an 18.0 keV monochromatic X-ray. A set of radiographs (720 projections over 180°) was recorded using an X-ray detector system with a 3.25-μm pixel size (ORCA-Flash4.0, Hamamatsu Photonics KK, and Japan). The slices were reconstructed using a filtered back-projection (FBP) algorithm, and 3D renderings were created and manipulated in VG Studio Max 2.1 software.

### Statistical analysis

Quantitative data are presented as the mean ± standard deviation and were analyzed using one-way ANOVA, followed by Tukey’s multiple-range test. *P* < 0.05 was considered statistically significant.

## Results

### Clinical and genetic identification of the patient

**Fig. 1:**
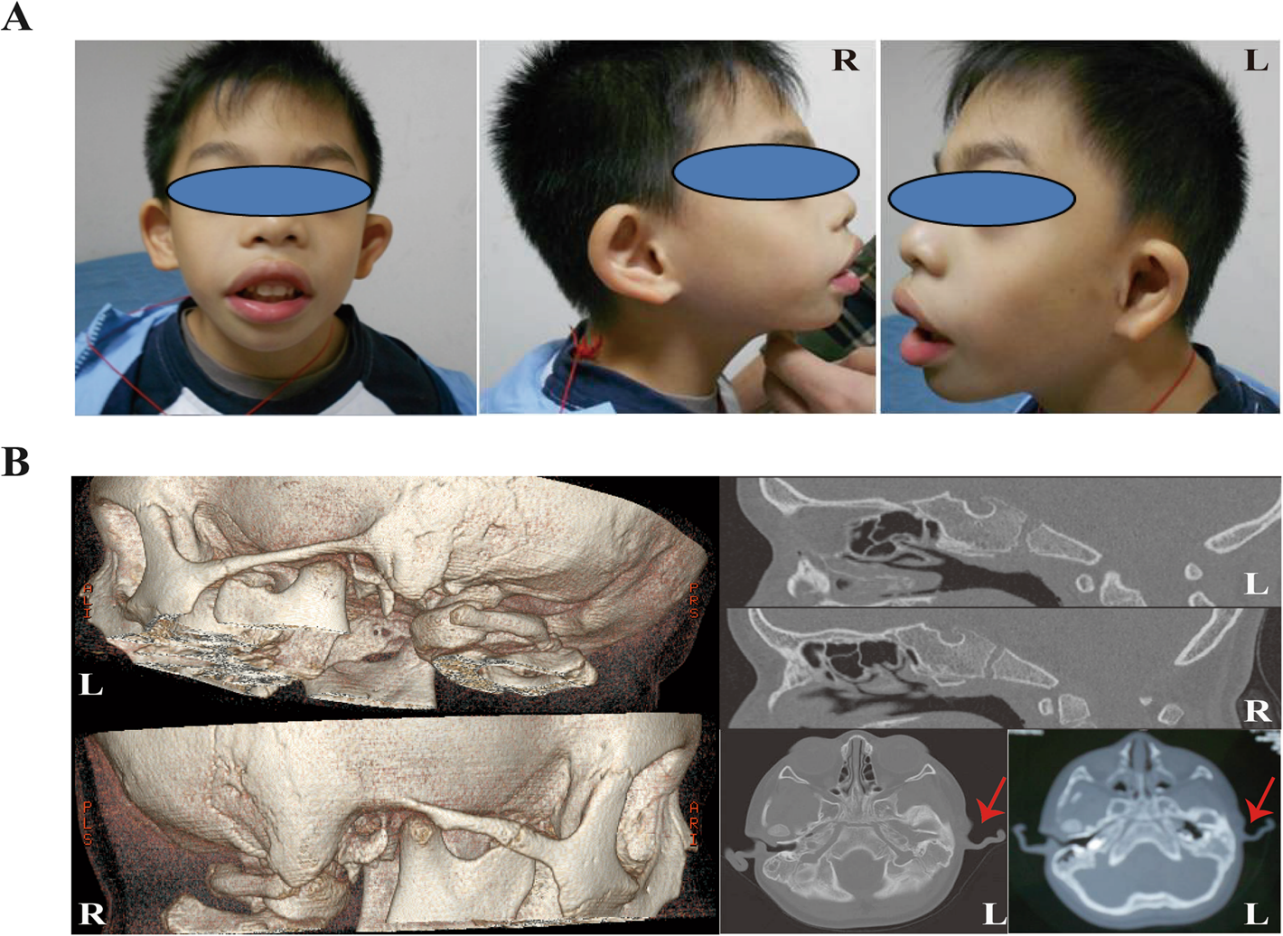
Clinical presentation of the proband. **a** Microcephaly, abnormal external ear, and mandibular hypoplasia features of the patient at 57 months of age. **b** Malformed structures of the external and middle ear on the left head and temporal bones via CT scan at 37 months of age; soft tissue could be seen in the left tympanic cavity. R, right; L, left

**Fig. 2:**
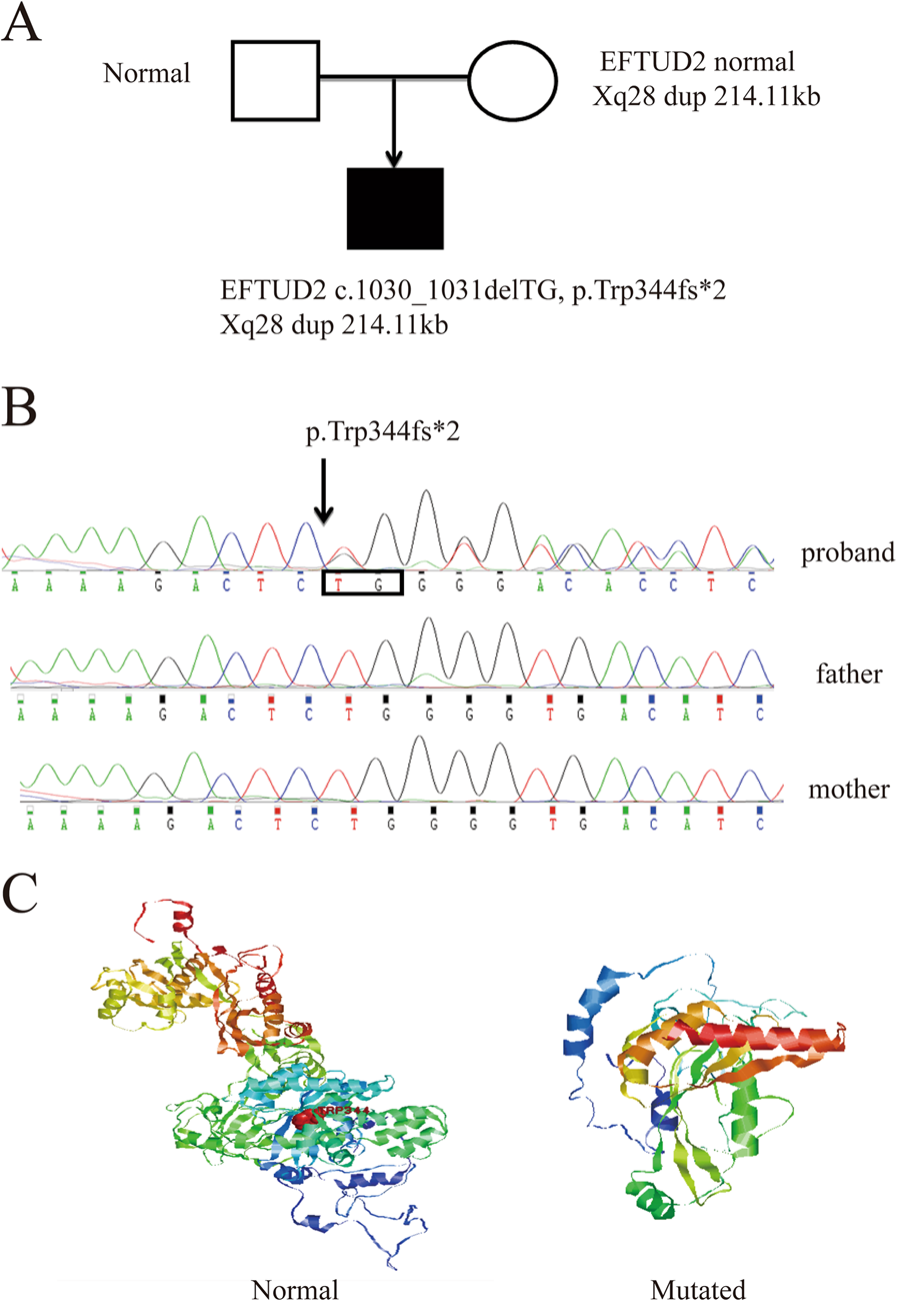
Pedigrees and EFTUD2 variant identified by family trio whole-exome sequencing. **a** Pedigree and genotype. **b** De novo heterozygous mutation c.1030_1031delTG (p.Trp344fs*2) in EFTUD2 (NM_001258353.1) in the proband. **c** Difference in protein structures between normal EFTUD2 and the variant

The proband was a G1P1 full-term boy with a birth weight of 2600 g, although the length was unavailable, who failed to pass the initial hearing test at 3 days and the second test at 42 days old in the left ear. The boy had a hoarse cry and poor sucking reflex after birth. He began to teeth at the age of 4 months, crawl at 7 months, pull self to a standing position at 9 months, and walked alone at 1 year of age, similar to other children of the same age. However, he walked unsteadily until approximately 25 months old and ran with poor coordination at 37 months. The child presented with microcephaly (head circumference of 45 cm, <P3), severe micrognathia, arched eyebrows, everted lips, broad nasal bridge, abnormal ear structures with hearing loss, adenoid hypertrophy, and physical growth delay (weight of 8.6 kg, <P3; length of 92.4 cm, P10–25) at 37 months of age (Fig. 1a) when he first visited our clinic. Adenotonsillectomy was performed, and he also received surgical operation due to poorly formed structures of the external and middle ear, with external auditory canal stenosis on the left (Fig. 1b); by the age of 57 months, boundary developmental delay was confirmed with an intelligence quotient of 61 (verbal IQ 60, performance IQ 69) via the WISC test. Preauricular tags, cleft palate, esophageal atresia, seizures, limb symptoms, and congenital heart defects were not observed. A mental development abnormality related to a 214.11-kb duplication in the chromosomal region Xq28 (151,557,556-151,771,665(hg19)) involving the *GABRA3*, *MIR105-1*, *MIR767*, and *MIR105-2* genes was found both in the proband and his normal mother using Array-CGH, while his father did not carry this variant. There was a case (Patient 250326) reported in DECEPHER with a 371-kb duplication within the region (151,532,990-151,904,036) who had conditions of delayed speech and language development and intellectual disability, and there were three reports derived from DGV by SNP array of cases carrying duplications of 197 kb (nssv655052), 222 kb (nssv657732), and 211 kb (nssv683872) [18]. A further WES test detected only one novel heterozygous out-of-frame deletion (c.1030_1031delTG, p.Trp344fs*2) in the *EFTUD2* gene (OMIM 610536), which was associated with developmental delay and craniofacial dysostosis in a biological context among 23 candidate genes by cascading filters through the Ingenuity tool, such as allele frequency below 0.01 from the available databases of 1000 Genomes, the NHLBI GO Exome Sequencing Project, and dbSNP (Fig. 2a, b and Additional file 1: Table S1). The variant truncated the wild-type 116 kDa *EFTUD2* protein into a protein of approximately 50 kDa, which is predicted to have a completely different protein structure than that of the wild type (Fig. 2c).

### Spatial and temporal expression of the *eftud2* gene during zebrafish development

Wild-type zebrafish larvae were sacrificed at 24 hpf, 48 hpf, 3 dpf, and 5 dpf, and *eftud2* gene expression was analyzed by whole mount in situ hybridization (WISH) and real-time PCR. *eftud2* was widely expressed throughout the embryo during early developmental stages (Additional file 2: Figure S1A). *eftud2* mRNA expression continued to increase until 48 hpf and decreased thereafter (Additional file 2: Figure S1B). Among the different tissues of adult zebrafish, the *eftud2* gene was expressed at high levels in the spleen, muscle, and brain, whereas it was expressed at a low level in ocular tissue (Additional file 2: Figure S1C).

### The efficacy of *eftud2* knockdown and knockout in zebrafish

**Fig. 3:**
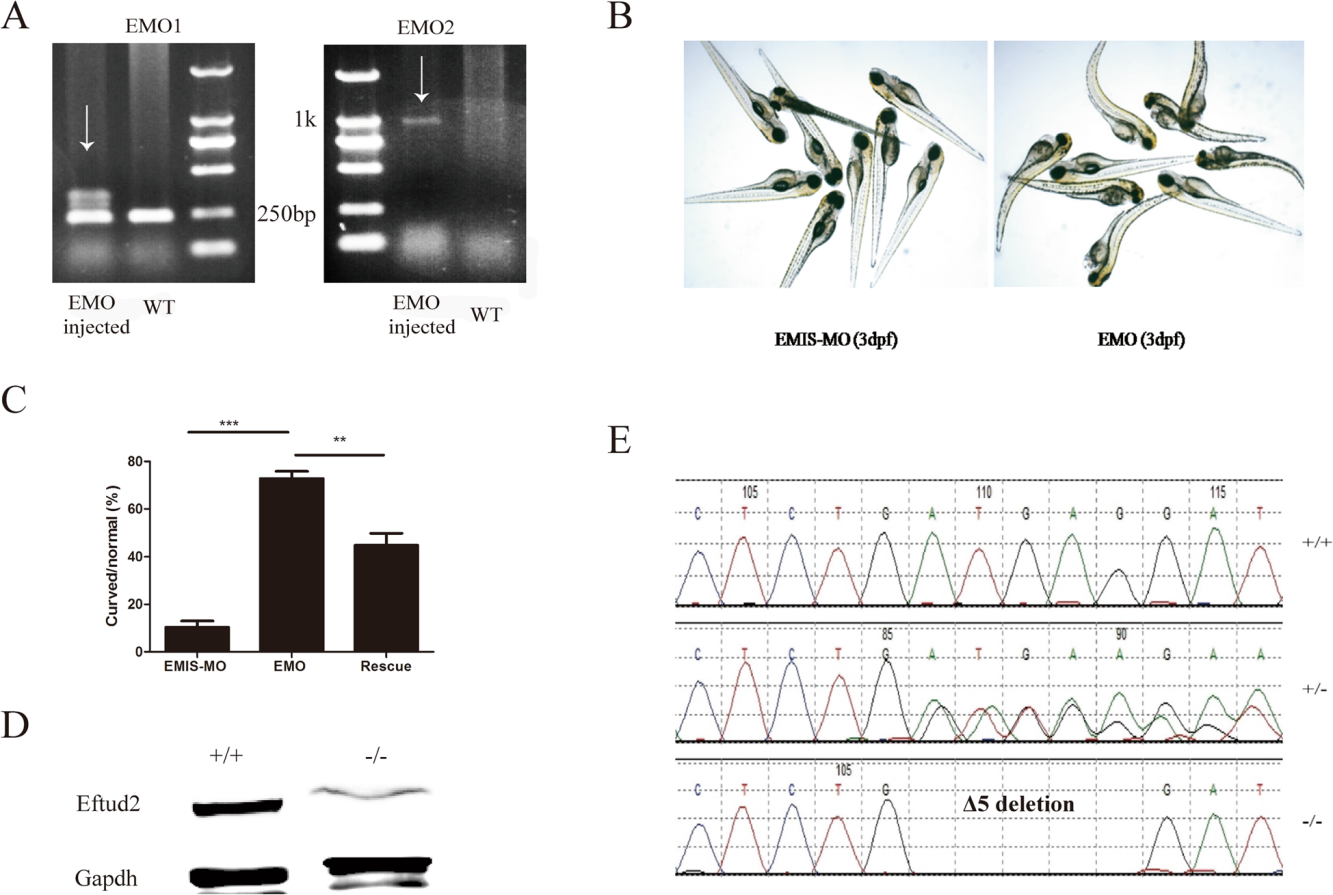
*eftud2* gene knockdown and knockout in zebrafish**. a** PCR analysis and gel electrophoresis to identify the efficacy of an *eftud2* morpholino (EMO). On the left, amplification was performed with the EMO1 primer, which resulted in a clear band in WT zebrafish and nonspecific bands in EMO-injected larvae. On the right, amplification was performed with the EMO2 primer, which produced a clear band in EMO-injected zebrafish and nothing in the WT. **b** Phenotypes of mutant zebrafish subjected to EMO injection and control morpholino (mismatch morpholino, EMIS-MO)-injected zebrafish at 3 dpf. **c** Comparison of the numbers of curved larvae among zebrafish injected with EMO, EMO together with normal EFTUD2 mRNA (rescue group), or EMIS-MO. **d** Western blot analysis of Eftud2 protein levels between the WT (+/+) and homozygous (−/−) mutant zebrafish at 3 dpf. **e** DNA sequencing of WT (+/+), heterozygous (+/−), and homozygous (−/−) mutants at 3 dpf

PCR results showed that EMO could affect the normal splicing of *eftud2*, thus leading to its abnormal expression. As EMO is designed to target the splicing site, DNA products amplified with EMO1 from EMO-injected zebrafish larvae at 48 hpf yielded nonspecific bands, except at 250 bp, and a large fragment (1 kbp) when amplified with EMO2 (Fig. 3a). Additionally, zebrafish with a disrupted *eftud2* gene exhibited curved bodies much more frequently than those injected with EMIS-MO or rescued with normal human *EFTUD2* mRNA (Fig. 3b, c).

To test the effect of long-term knockout of the *eftud2* gene, targeted knockout TALENs (Additional file 3: Figure S2A, B) were established and selected through in vitro luciferase activity (Additional file 3: Figure S2C), T7E1 enzyme digestion (Additional file 3: Figure S2D), DNA sequencing (Additional file 3: Figure S2E), and Western blot assays (Fig. 3c). The successful *eftud2* knockout model was confirmed via Sanger sequencing of zebrafish larvae at 3 dpf, which showed a deletion of 5 bp (c.59-63 ATGAG), and Western blot analysis showed sharply decreased protein expression (Fig. 3d, e). The deletion of 5 bp (Δ5) resulted in a frameshift mutation that translated to a shortened EFTUD2 protein containing only the first 19 amino acids when compared with the reference amino acids (XP_017209079.1).

### *eftud2* knockdown and knockout disrupted bone and cartilage development in zebrafish

Short-term knockdown of the *eftud2* gene with a specific morpholino (EMO) resulted in a significantly higher rate of curved bodies (72 ± 5.14%) compared with that of the EMIS-MO control group at 3 dpf (10.3 ± 4.6%, *P* < 0.00, Fig. 3b, c). The coinjection of EMO with normal human *EFTUD2* mRNA, which shares 91% nucleotide homology with that of zebrafish, was employed as a rescue intervention. Rescue in EMO-treated zebrafish significantly decreased the incidence of curved larvae to 44.9 ± 8.8% (*P* < 0.01, Fig. 3c). Dysplasia of Meckel’s cartilage and ceratohyals was also observed in the larvae that received an injection of EMO at 3 dpf and 5 dpf, based on alcian blue or alizarin red staining, and the coinjection of EMO and normal human *EFTUD2* mRNA could also rescue the dysgenesis in mandibular bone (Additional file 4: Figure S3A–D).

**Fig. 4:**
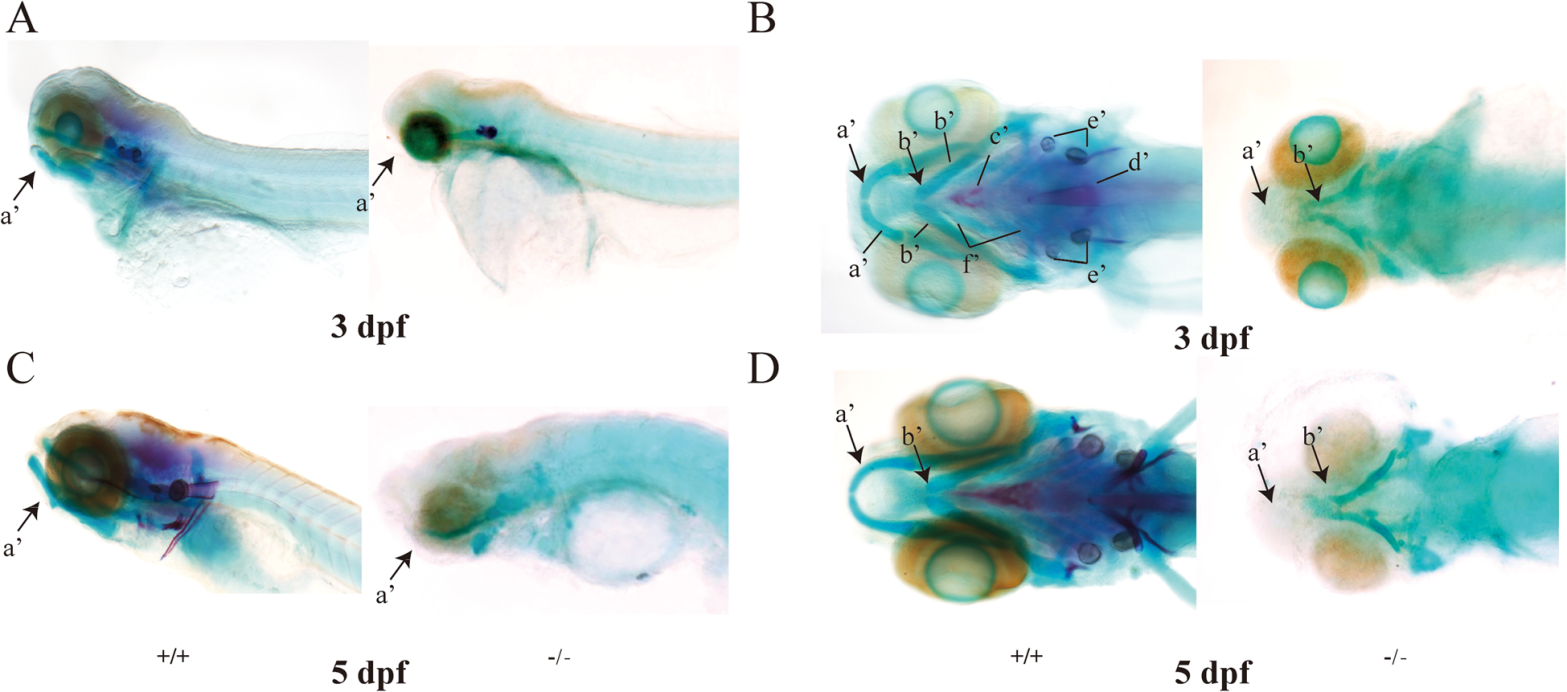
Zebrafish subjected to *eftud2* gene knockout had aberrant cartilage and bone development. **a**, **c** Lateral view of larvae treated with *eftud2* TALEN mRNA (−/−) at 3 dpf and 5 dpf, exhibiting disruption of cartilage and bone formation compared with WT (+/+). **b**, **d** Ventral view of larvae treated with *eftud2* TALEN mRNA (−/−) at 3 dpf and 5 dpf, showing cartilage and bone hypoplasia and otolith loss compared with WT (+/+). (The images on the left are WT controls, and those on the right are *eftud2* mutants. Black arrows indicate the jawbone. **a**’ Meckel’s bone. **b**’ Ceratohyal bone. **c**’ Parasphenoid. **d**’ Notochord. **e**’ Otolith. **f**’ Ceratobranchial 1–5)

**Fig. 5:**
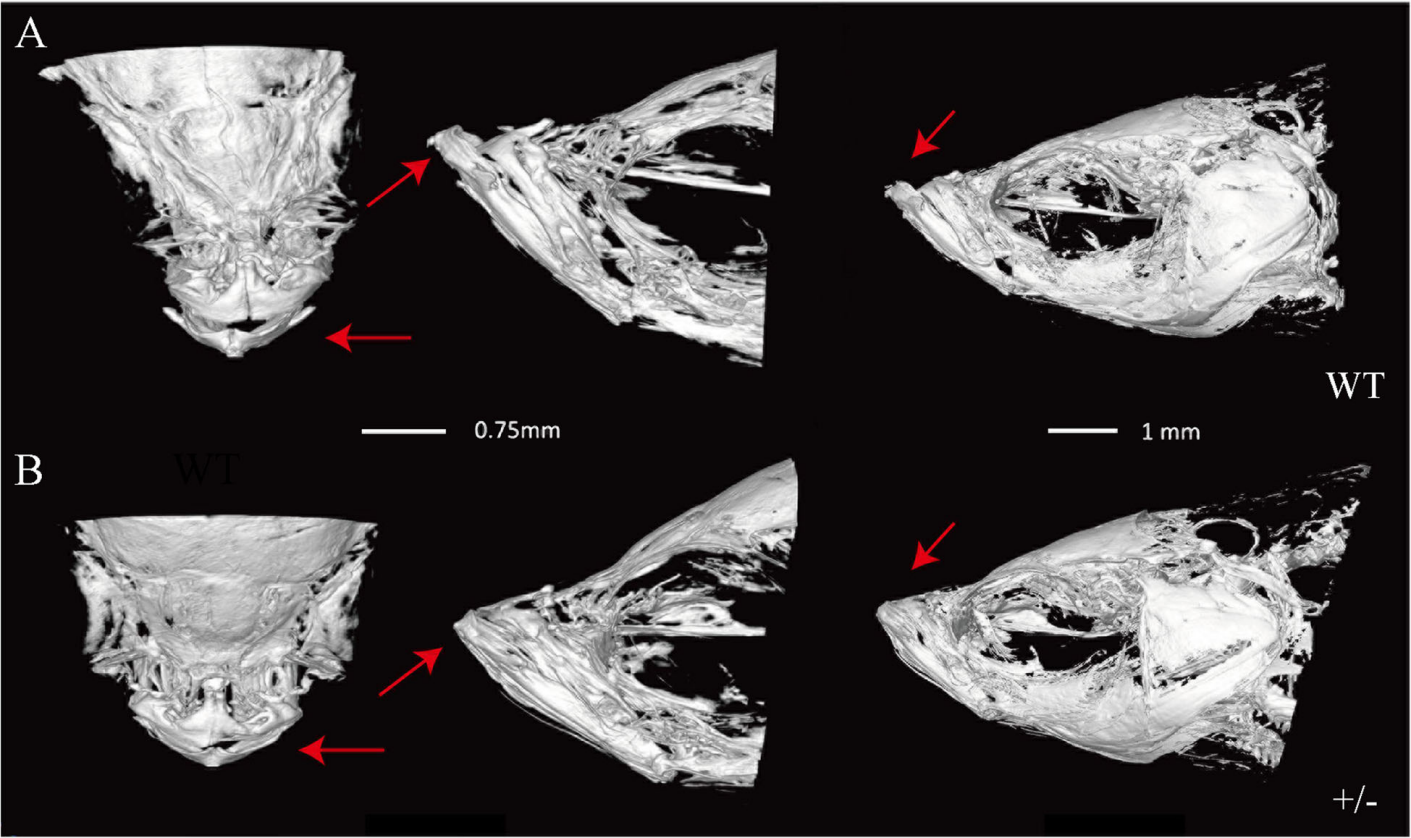
Adult zebrafish with heterozygous *eftud2* mutation showed abnormal mandibular bone. **a** Mandible bone of wild-type adults (WT) scanned by synchrotron radiation X-ray microtomography (arrows). Scale bar, 0.75 mm. **b** The heterozygous F2 generation (+/−) exhibited a shortened mandibular bone (arrows). Images on the left are viewed from the dorsal side, while the images in the middle and on the right show lateral views. Scale bar, 1 mm

In addition to small heads and large yolks, the stable *eftud2* TALEN knockout zebrafish showed obviously significant dysplasia formation in their Meckel’s cartilage, ceratohyals, and ethmoid bones compared with the larvae injected with EMO at 3 dpf and 5 dpf (Figs. 4 and 5). Malformations in the sphenoidal sinus and notochord and even otolith loss were also recorded in the knockout larvae (Fig. 4a–d). Interestingly, all of the larvae with homozygous *eftud2* mutation died within 5 days; therefore, only the adult zebrafish with heterozygous *eftud2* mutation were available for phenotypic and genotypic analyses. The heterozygous F2-generation knockout adults exhibited a phenotype much more similar to that of human MFDM, presenting a shortened jawbone and deformity of Meckel’s cartilage by synchrotron radiation X-ray microtomography (Fig. 5a, b).

### *EFTUD*2 gene knockdown suppressed HCO and HC-a cell growth in vitro

**Fig. 6:**
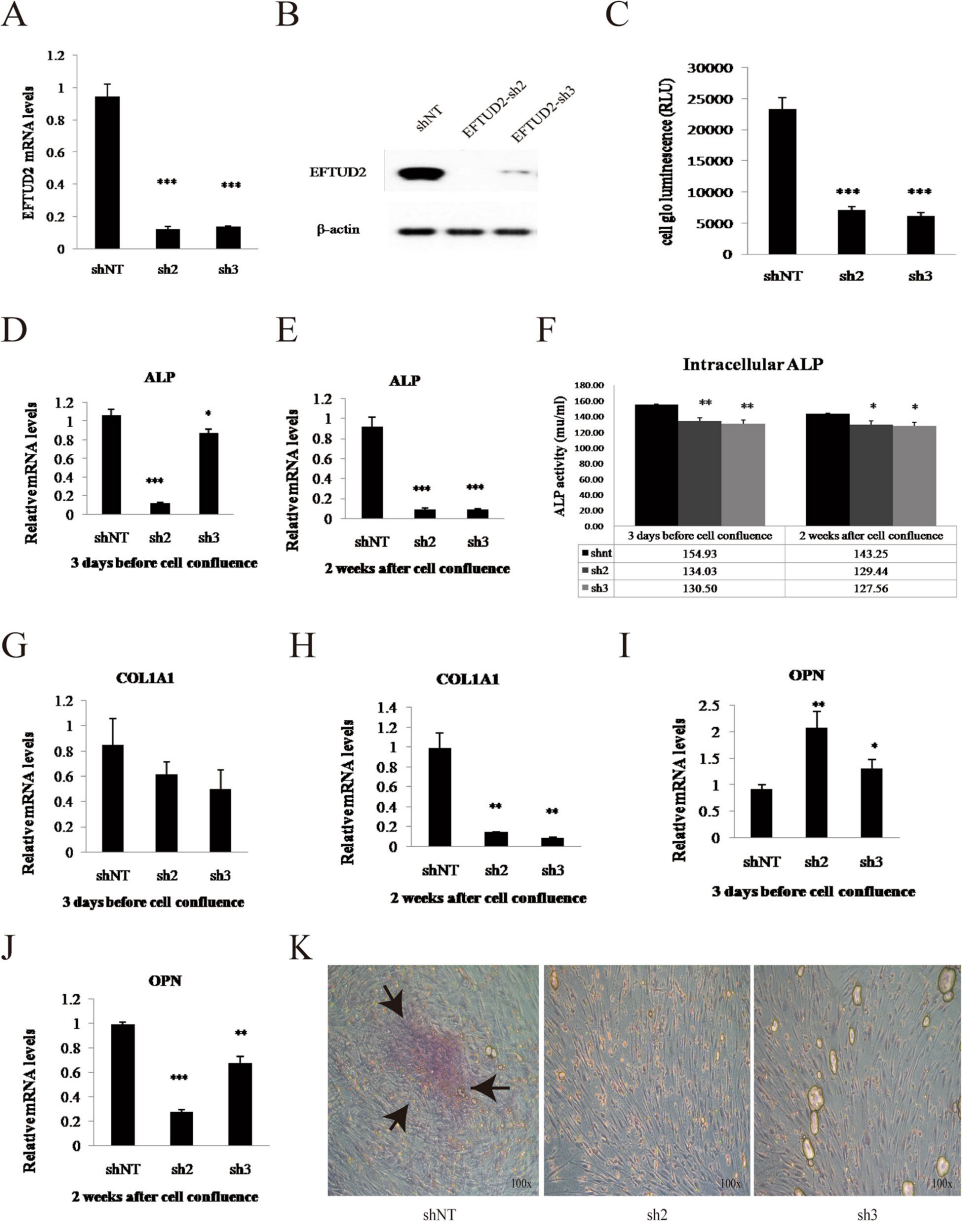
*EFTUD2* gene knockdown in HCO cells. **a** Expression of *EFTUD2* mRNA in HCO cells transfected with shNT, sh2, and sh3 lentiviruses. **b** Protein expression of *EFTUD2* in HCO cells transfected with shNT, sh2, and sh3 lentiviruses. **c** Comparison of cell proliferation between HCO cells with EFTUD2 knockdown (sh2 and sh3) and the control group (shNT). **d**, **e** ALP mRNA levels in different groups (shNT, sh2, sh3) at 3 days before and 2 weeks after cell confluence in HCO cells. **f** Intracellular ALP activity (mu/ml) in HCO cells with *EFTUD2* knockdown (sh2 and sh3) was much lower than in the control (shNT) at 3 days before cell confluence according to Student’s *t* test. **g**, **h** COL1A1 mRNA levels in HCO cells in different groups (shNT, sh2, sh3) at 3 days before and 2 weeks after cell confluence. **i**, **j** OPN mRNA levels in different groups (shNT, sh2, sh3) of HCO cells during development**. k** Alizarin red staining of HCO cells in these 3 groups at 3 days after cell confluence. Black arrows indicate calcific nodules (*P < 0.05; **P < 0.01; and ***P < 0.001; Student’s *t* test)

To determine whether the *EFTUD2* gene affects osteoblast proliferation, HCO cells were transfected with targeting shRNAs (sh2 and sh3, Fig. 6a–c). HCO cell proliferation decreased by 65% after lentivirus transfection (*P* < 0.0001) compared with that after shNT treatment at 3 days before cell confluence (Fig. 6c and k). Furthermore, the expression of relevant genes, including the alkaline phosphatase (*ALP*), collagen type 1 alpha 1 (*COL1A1*), and osteopontin (*OPN*) genes (Fig. 5d, e, g, j), along with ALP activity (Fig. 6f), was significantly decreased throughout the proliferation and differentiation period (*P* < 0.01). COL1A1, which is a marker of the early differentiation and maturation of the extracellular matrix, showed no change before cell confluence and was decreased by 90% when entering the differentiation phase in the sh2 and sh3 groups compared with that in the control group (*P* < 0.01) (Fig. 6g, h). The expression levels of *OPN*, a marker of mineralization during the late differentiation of the cellular matrix, in HCO cells transfected with sh2 and sh3 were 2.3- and 1.4-fold higher (*P* < 0.01 and *P* < 0.05) than that in shNT-treated cells at 3 days before cell confluence, suggesting that HCO cells mature early in the differentiation stage (Fig. 6i). Thereafter, *OPN* expression declined by 30% and 70% (*P* < 0.001, *P* < 0.01) in the sh2 and sh3 groups, respectively, indicating the inhibition of differentiation in the late differentiation stage (Fig. 6j). Calcium deposition significantly and consistently decreased without evident calcific nodules in those *EFTUD2* knockdown groups (Fig. 6k).

We also investigated the role of *EFTUD2* in HC-a cell proliferation via *EFTUD*2-targeting shRNAs (sh2 and sh3, Additional file 5: Figure S4A–C). Similar to HCO cells, HC-a cell proliferation was significantly downregulated by approximately 70% (*P* < 0.001) (Additional file 5: Figure S4C and S4H) 3 days before cell confluence. The expression levels of differentiation-related genes in HC-a cells, including the collagen type 10 alpha 1 (*COL10A1*) and SRY-box 9 (*SOX9*) genes, were assayed; *COL10A1* gene expression was observed to be significantly increased by approximately 2-fold before cell confluence, after which it decreased at the differentiation stage (Additional file 5: Figure S4D, E). *SOX9*, the key transcription factor throughout cartilage formation, was significantly reduced throughout the proliferation and differentiation stages (Additional file 5: Figure S4F, G).

### *EFTUD2* knockdown and knockout resulted in *TP53* pathway activation in vitro and in vivo

**Fig. 7:**
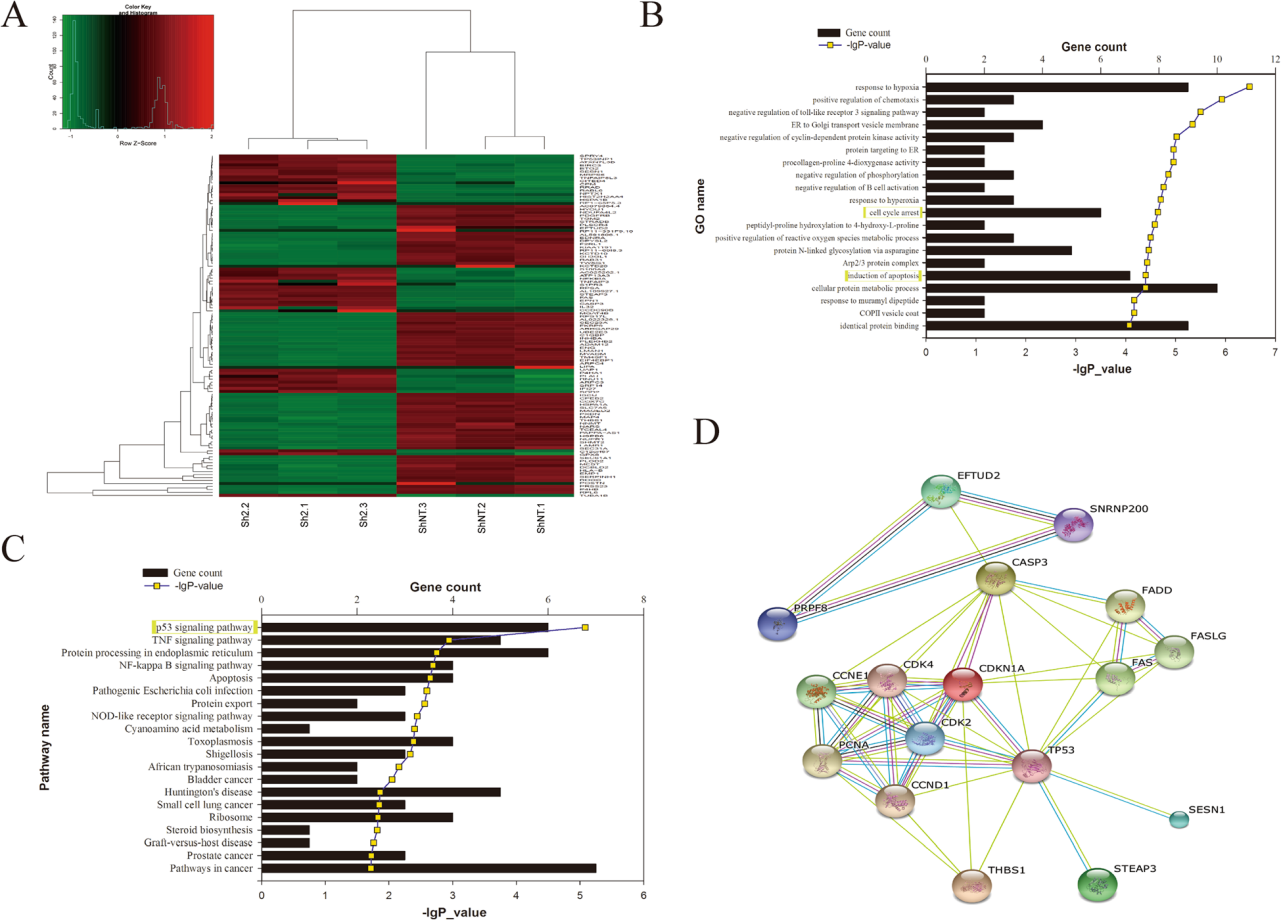
RNA-Seq analysis in HCO cells. **a** Hierarchical cluster heatmap of *Z*-scores for relevant genes between the sh2 and shNT groups, each of which includes three samples (sh2-1, sh2-2, sh2-3; shNT-1, shNT-2, shNT-3). **b** GO annotation revealed the most significant enrichment (*P* < 0.001), among which “cell cycle arrest” and “induction of apoptosis” were directly associated with cell development. **c** KEGG metabolic pathway categories with a significant *P* value; the P53 pathway was predicted to be the most likely (*P* < 0.001). **d** Protein interactions between EFTUD2 and the P53 pathway determined by the STRING website

A total of 997 differentially expressed genes were identified by RNA sequencing in HCO cells (Fig. 7a and Additional file 6: Figure S5A, B), and 20 genes showing the closest relationships were selected through GO and KEGG enrichment analysis (Fig. 7b, c). The cell cycle arrest and apoptosis genes were the most strongly affected in HCO cells after *EFTUD2* gene disruption (Fig. 7b), while the *TP53* pathway was suggested to be the most significant pathway according to KEGG analysis (Fig. 7c). Among the 6 genes involved in the *TP53* (Tumor protein p53) downstream pathway, 5 genes, including *FAS* (Fas cell surface death receptor), *STEAP3* (STEAP3 metalloreductase), *CASP3* (Caspase-3), *P21* (Cyclin-dependent kinase inhibitor), and *SESN1* (STEAP3 metalloreductase), were upregulated, whereas the *THBS* (Sestrin 1) gene was downregulated (Additional file 6: Figure S5C). STRING Protein-Protein Interaction Network analysis suggested that the *EFTUD2* gene could affect cell growth through *CASP3* (Fig. 7d).

**Fig. 8:**
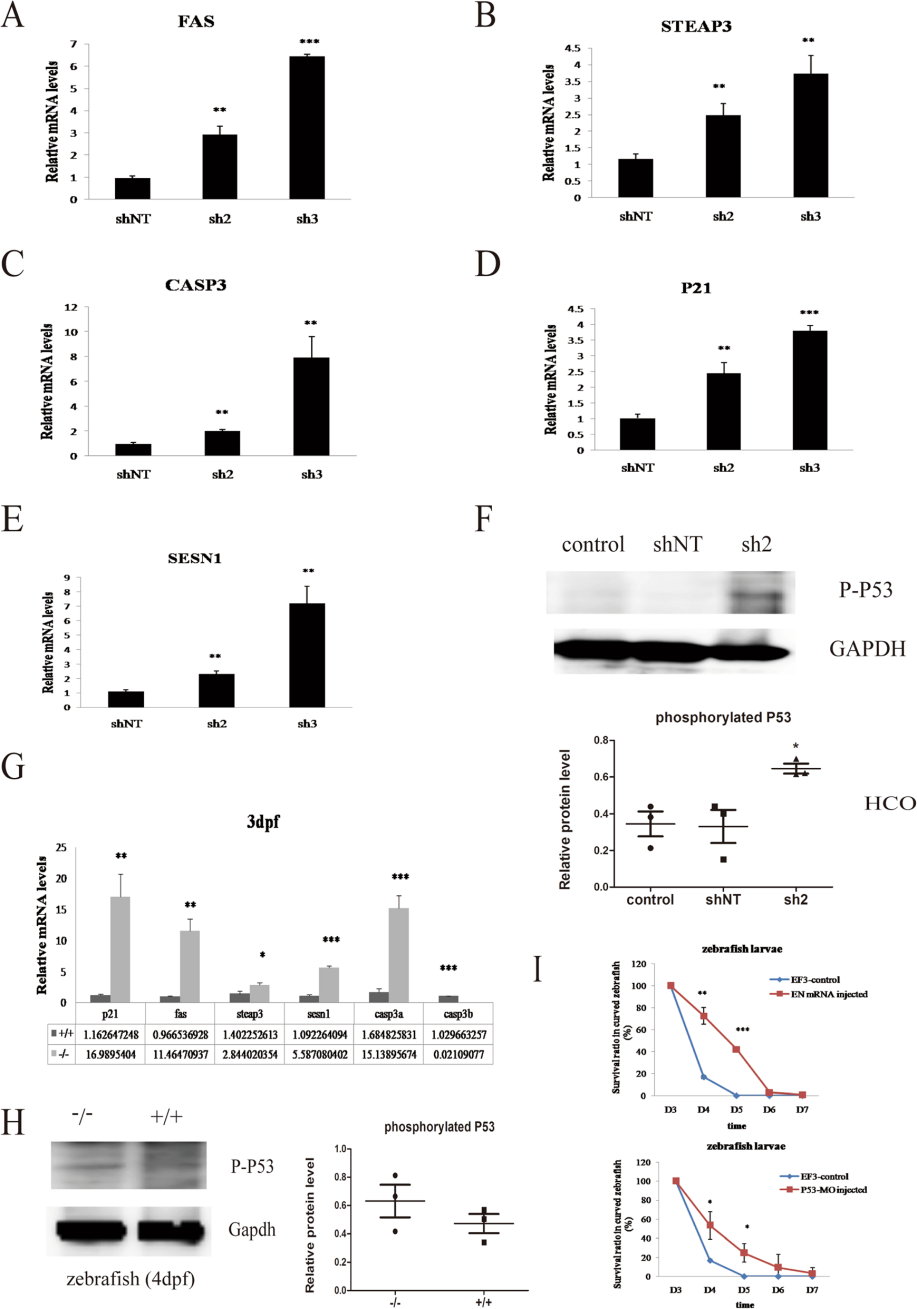
The p53 pathway mediates cell development in HCO cells and zebrafish. **a** Expression of the *FAS* gene in cells transfected with different lentiviruses (sh2, sh3, and shNT as a scramble control) at 3 days before cell confluence. HCO cells subjected to EFTUD2 knockdown (sh2 and sh3) exhibited increased *FAS* levels. **b** The expression of *CASP3* in the sh2 and sh3 groups was dramatically increased compared with that in the shNT group. **c** *STEAP3* mRNA levels in the sh2 and sh3 groups were markedly higher than that in the negative control. **d** *P21* mRNA levels were also highly increased in the sh2 and sh3 groups. **e** *SESN1* was highly expressed in the sh2 and sh3 groups. **f** HCO cells transfected with sh2 had a higher expression of phosphorylated P53 protein (53 kDa) than the nontransfected (control) and shNT groups. **g** Expression of relevant genes involved in the P53 pathway in the EFTUD2 knockout (−/−) and WT (+/+) zebrafish. **h** The expression of phosphorylated P53 in *EFTUD2*-mutated larvae (−/−) with curved bodies and WT larvae at 4 dpf. **i** The survival rate among the curved F3 generation hybridizing from *EFTUD2* heterozygous mutants (EF3 control), EF3 controls injected with EFTUD2 normal mRNA (EN mRNA), and p53 morpholino (P53-MO) during the early developmental stage (*P < 0.05, **P < 0.01, and ***P < 0.001)

We then analyzed the profiles of *FAS*, *STEAP3*, *CASP3*, *P21*, and *SESN1* after *EFTUD2* gene intervention and observed a significant increase of approximately 1.9- to 6.7-fold over baseline for *FAS*, *CASP3*, *STEAP3*, *P21*, and *SESN1* at 3 days before cell confluence in HCO cells (Fig. 8a–e). HCO cells with *EFTUD*2 knockdown (sh2) had higher protein levels of phosphorylated P53 compared with the shNT-transfected and nontransfected (control) groups (*P* < 0.05, Fig. 8f), whereas there was no difference between the negative control groups (shNT and control). Compared with that in the WT sample at 3 dpf, the expression of the *P21*, *FAS*, *STEAP3*, *SESN1*, and *CASP3A* genes in the *p53* pathway was 2- to 14.6-fold higher (*P* < 0.01, *P* < 0.01, *P* < 0.05, *P* < 0.001, and *P* < 0.001, respectively) in the homozygous *eftud2* mutant zebrafish (Fig. 8g). *CASP3B* expression decreased to 2% of the WT expression level (*P* < 0.001). The protein expression of phosphorylated P53 (P-P53) was slightly elevated in the mutants with curved bodies at 4 dpf (*n* = 20, *P* = 0.51, Fig. 8h), which may result from protein degradation. For further validation, we injected the *p53* morpholino into F3 generation (EF3) hybrids from *eftud2* heterozygous mutated zebrafish (F2) and found that P53 knockdown could decrease the mortality of those curved larvae at 4 dpf and 5 dpf (*P* < 0.05), similar to those with normal human *EFTUD2* mRNA injected to a certain extent (*P* < 0.01 and *P* < 0.001, Fig. 8i).

## Discussion

By WES, a novel de novo frameshift *EFTUD2* gene variant (c.1030_1031delTG, p.Trp344fs*2) was found in the first Chinese mandibulofacial dysostosis, Guion-Almeida type (MFDGA; MIM #610536) patient with distinctive facial features, including arched eyebrows, broad nasal bridge, everted lips and micrognathia, low-set ears and external auditory canal stenosis, mild intellectual disability, and developmental delay. A 214.11-kb duplication in the chromosomal Xq28 region including the GABRA3, MIR105-1, MIR767, and MIR105-2 genes, identified in the proband via microarray analysis, was inherited from his healthy mother. Evidence from DGV indicated duplications of 197 kb (nssv655052), 222 kb (nssv657732), and 211 kb (nssv683872); these duplications were interpreted as likely benign, though a case (Patient 250326) reported in DECEPHER with a 371-kb duplication within the region had conditions of delayed speech, language development, and intellectual disability but without facial dysmorphological features [18]. Thus, the de novo deletion of TG resulting in the frameshift variant of *EFTUD2* identified in the patient by WES would be pathogenic.

The widespread expression of *eftud2* in mice at 11.5 dpf was previously observed to correlate with the affected region in patients [12], but there has been no description of *eftud2* expression in the zebrafish model. We observed a wide *eftud2* expression pattern in zebrafish embryos at 24 hpf, and expression continued increasing until 48 hpf. In addition to small heads and curved bodies in the early development stage in *eftud2−/−* zebrafish larvae, which was consistent with a previous report [13], our knockout zebrafish model demonstrated other extra characteristics, including the absence of otoliths as well as bone and cartilage abnormalities. Synchrotron radiation X-ray microtomography showed that adult heterozygous F2-generation zebrafish exhibited a shortened jawbone and deformity of Meckel’s cartilage, which mimic the phenotypes of human MFDM, suggesting the pathogenesis of *EFTUD2* correlated with the craniofacial skeleton.

Craniofacial bone is formed by endochondral and intramembranous ossification. We generated *EFTUD2* knockdown in two cell lines, HCO cells and HC-a cells, to identify its influence on osteoblast proliferation. We observed a significant reduction in cell proliferation in the two cell lines. The expression of *ALP* and *COL1A1*, early differentiation markers [19, 20], was significantly decreased during the differentiation period in HCO cells. Furthermore, *OPN*, a marker of mineralization during the late differentiation of the cellular matrix, also reduced its expression by a maximum of 70%. In addition, decreased calcium deposition and the formation of calcified nodules were found in HCO cells following *EFTUD2* shRNA treatment, which highly suggests that HCO cell differentiation was prematurely stopped. *Sox9*, a key transcription factor throughout the formation of cartilage [21–24], also presented a significant reduction throughout the proliferation and differentiation stages in HC-a cells. These findings demonstrated that *EFTUD2* knockdown could dramatically decrease the proliferation and inhibit the differentiation of HCO and HC-a cells. *EFTUD2* may play a role in maintaining normal calvarial osteoblast and chondrocyte proliferation.

High-throughput RNA sequencing revealed the *TP53* signaling pathway was most strongly affected, and cell cycle arrest and apoptosis induction were significantly affected in *EFTUD2* knockdown HCO cells. *TP53*, which is an important tumor suppressor gene, is responsible for cell growth, including the cell cycle, apoptosis, and angiogenesis [25, 26]. A further experiment found increased phosphorylation of the TP53 protein, suggesting activation of the *TP53* signaling pathway, which was validated in the knockout zebrafish model (4 dpf). Additionally, *p53* knockdown definitely increased the survival of those mutated zebrafish larvae.

Furthermore, we identified increased expression in 5 out of the 6 major *TP53*-downstream cluster genes (*FAS*, *STEAP*3, *CASP3*, *P21*, and *SESN1*) in HCO cells, which was also validated in homozygotic *eftud2* knockout zebrafish. Previous studies have shown that the *TP53* signaling pathway is involved in inherited diseases with maxillofacial signs, such as Treacher Collins syndrome (TCS) or Acrofacial Dysostosis-Cincinnati type [27–29], neural stem cell apoptosis during embryonic development [30], and *TP53*-dependent apoptosis in neural progenitors in fn10a-mutated zebrafish [31]. Deml et al. reported a striking rise in apoptosis in many tissues in an *eftud2*-deficient zebrafish model [13]. Therefore, evidence of increasing *TP53* signaling pathway activation was correlated with subsequent cell apoptosis, which could impact development, particularly ossification.

## Conclusions

We identify a novel de novo frameshift *EFTUD2* gene variant (c.1030_1031delTG, p.Trp344fs*2) in a Chinese MFDM patient. We established an *EFTUD2* deficiency model in vitro and in vivo. Evidence of the transcriptome from cell lines and a zebrafish model suggested the *TP53* signaling pathway was activated due to *EFTUD2* disruption. Our findings also showed that the *EFTUD2* gene could impact the proliferation and differentiation of osteoblasts and chondrocytes, suggesting that premature osteoblast and chondrocyte differentiation could be responsible for the pathogenesis of MFDM. Further studies on the specific mechanisms involved are necessary in the future.

## Supplementary information

**Additional file 1. **Materials and Methods including whole-genome aCGH analysis, whole-exome sequencing, embryo injection, luciferase single-strand annealing recombination analysis, DNA extraction and PCR for sequencing, real-time PCR and Western blotting. **Table S1.** Candidate genes with *de novo* variants in the proband. **Table S2.** Real-time PCR primers of relative genes in cells.

**Additional file 2: Figure S1.** *eftud2* expression pattern in WT zebrafish. A: *eftud2* expression in zebrafish embryos at 24hpf (a, b), 48hpf (c, d), 3dpf (e, f) and 5dpf (g, h) was examined using whole-mount *in situ* hybridization over a period of 5 days, employing a specific *eftud2* anti-sense probe; (a, c, e, g) lateral view, (b, d, f, h) dorsal view. B: Relative mRNA levels of *eftud2* during the early developmental stages. C: Relative mRNA levels of *eftud2* in adult zebrafish tissues.

**Additional file 3: Figure S2.** Targeting site and efficacy of *eftud2*-targeted TALEN mRNAs. We also prepared three TALENs to construct the knockout zebrafish model, and the second one (T2) was the most effective for *in vitro* screening. A, B: The TALEN (T2) was designed at the first exon, which is presented in capital letters. C: *In vitro* efficacy was evaluated based on relative luciferase activity in TALEN-transfected Hek293T cells and the negative control. D: PCR products of *eftud2* containing the target sequence could be digested by the T7E1 enzyme, in which the product from the mutant zebrafish was cleaved into two fragments, whereas that from WT zebrafish was intact. E: Sequencing results of F0 generation showed mixed signals from the target site, which may predict the combination with TALEN mRNA and the *eftud2* gene.

**Additional file 4: Figure S3.** Zebrafish with *eftud2* gene knockdown showed aberrant cartilage development. A, B: Larvae treated with an *eftud2* morpholino (EMO) at 3dpf exhibited disrupted formation of Meckel’s cartilage (a) and the ceratohyals (b) upon alcian blue and alizarin red staining compared with the WT fish, fish injected with a mismatch morpholino (EMIS-MO) and fish rescued with normal human EFTUD2 mRNA (Rescue). A shows the lateral view, and B shows the ventral view. C, D: Bone and cartilage staining among different groups of larvae (WT, EMO, rescue, EMIS-MO) also suggested abnormal cartilage development at 5dpf.c, the ethmoid bones.

**Additional file 5: Figure S4.** *EFTUD2* gene knockdown in HC-a. A: Expression of *EFTUD2* mRNA in HC-a cells transfected with sh2 and sh3 lentivirus was lower than that in the shNT control.B: Protein expression of *EFTUD2* decreased in HC-a cells transfected with sh2 and sh3 lentivirus. C: Cell proliferation of HC-a cells transfected with sh2 and sh3 lentivirus was disrupted compared with that of the shNT control. D-E: *COL10A1* mRNA levels among different groups (shNT, sh2, sh3) of HC-a cells at 3 days before cell confluence, 3 days after cell confluence and 2 weeks after cell confluence. F-G: *SOX9* mRNA levels in HC-a cells among different groups (shNT, sh2, sh3) at 3 days before and after cell confluence. H: Alcian blue staining of HC-a cells among different groups (transfected with shNT, sh2 and sh3 lentiviruses). *: *P* < 0.05, **: *P* < 0.01, ***: *P* < 0.001.

**Additional file 6: Figure S5.** Differentially expressed genes identified through RNA-Seq analysis. A: Correlation analysis between HCO cells transfected with shNT (Group1) or sh2 lentivirus (Group2), which showed a close relationship between the two groups. B: Genes of Group 1 and Group2 are located on all of the chromosomes. C: There were 6 genes involved in the P53 pathway, including *P21*, *FAS*, *STEAP3*, *CASP3*, *SESN1* and *THBS*. All of these genes showed elevated expression, except *THBS*, which was downregulated.

## Data Availability

The datasets used and/or analyzed during the current study are available from the corresponding author upon reasonable request.

## Abbreviations

a-CGH: Array-CGH
ATP: Adenosine triphosphate
CASP3: Caspase-3
COL10A1: Collagen type 10 alpha 1
COL1A1: Collagen type 1 alpha 1
CT: Computerized tomography scan
DEGs: Differentially expressed genes
EF3: Hybridizing from heterozygous mutated zebrafish of F2-generation
EFTUD2: Elongation factor Tu GTP-binding domain-containing 2
EMIS-MO: *eftud2* mismatch control morpholino
EMO: *eftud2* morpholino
F1: Out-crossed F0 mosaic with wild-type zebrafish
F2: Obtained from *eftud2* heterozygous mutants
FAS: Fas cell surface death receptor
FBP: Filtered back-projection
GO: Gene ontology
HC-a: Human articular chondrocyte
HCO: Human calvarial osteoblast
KEGG: Kyoto Encyclopedia of Genes and Genomes pathway
MFDM: Mandibulofacial dysostosis with microcephaly
MUP: 4-Methylumbelliferyl phosphate disodium salt
OPN: Osteopontin
P21: Cyclin-dependent kinase inhibitor
PBS: Phosphate buffer saline
PFA: Paraformaldehyde
P-P53: Phosphorylated P53
SESN1: STEAP3 metalloreductase
shNT: Scrambled shRNA
SNVs: Single nucleotide variants
SOX9: SRY-box 9
SR-μCT: Synchrotron radiation X-ray microtomography
SSRF: Shanghai Synchrotron Radiation Facility
STEAP3: STEAP3 metalloreductase
STRING: Retrieval of Interacting Genes
TALEN: Transcription activator-like effector nucleases
THBS: Sestrin 1
TP53: Tumor protein p53
WES: Whole-exome sequencing
WISC: Wechsler Intelligence Scale for Children
WISH: Whole mount in situ hybridization
WT: Wild type
