# Supplementary material for: EFTUD2 gene deficiency disrupts osteoblast maturation and inhibits chondrocyte differentiation via activation of the p53 signaling pathway
Source: Hum Genomics. 2019 Dec 5;13:63. doi: 10.1186/s40246-019-0238-y (PMC6894506; doi:10.1186/s40246-019-0238-y)
Supplement: Supplementary file 1 — Additional file 1. Materials and Methods including whole-genome aCGH analysis, whole-exome sequencing, embryo injection, luciferase single-strand annealing recombination analysis, DNA extraction and PCR for sequencing, real-time PCR and Western blotting. Table S1. Candidate genes with de novo variants in the proband. Table S2. Real-time PCR primers of relative genes in cells. [file 40246_2019_238_MOESM1_ESM.docx]

# Additional files

## Materials and Methods

**Whole-Genome aCGH analysis**

We performed array-CGH Agilent 180K (Agilent Technologies, Shanghai, China) for copy number variation (CNV) detection of the proband and his parents. The experiment followed the manufacturer’s protocol, and data analysis of CNVs was carried out by Cytogenomics 2.0 (Agilent technologies, Shanghai, China) with GRCh37/hg19. The CNVs were evaluated by the DGV database (http://dgv.tcag.ca/dgv/app/home) and DECIPHER or OMIM, which refers to the public UCSC browser (https://genome.ucsc.edu/).

**Whole-exome sequencing**

The whole-exome sequencing approach was performed to identify the disease-associated genes in the proband and his parents by Claritas Genomics. A total of 3 µg of genomic DNA sample was randomly fragmented by a CovarisS2 Ultrasonicator (Covaris, MA, USA), and the fragments with a size between 150 bp and 200 bp were collected. With the ligated adapters, a paired-end library was prepared using the Sequencing Library Prep Kit (Agilent Technologies, CA, USA).Whole-exome targets were captured using the Agilent Sure Select Human All Exon V4 Kit (Agilent Technologies, CA, USA) following the vendor-provided protocols. Fifty-megabase target regions were covered (CCDS exonic regions and flanking intronic regions). The quality and size range of captured DNA was assessed by an Agilent 2200 Bioanalyzer with a high-sensitivity DNA kit (Agilent Technologies, CA, USA). The quantity of each index-tagged library was determined using a Q-PCR NGS Library Kit (Agilent Technologies, CA, USA) and Qubit® 2.0 Fluorometer (Life Technologies, Carlsbad, CA). Then, we performed multiplex sequencing with paired 100x100 base pair reads on an Illumina HiSeq System 2000 (Illumina, CA, USA).

## Embryo injection

For EFTUD2 knock-down analysis, morpholinos with or without normal human EFTUD2 mRNA (EN) were injected separately (EMO, EMIS-MO and EMO+EN). For *eftud2* mutant lines, targeted TALEN mRNAs (100ng/μl) were injected into zebrafish embryos at the one-cell stage using a Picospritzer injector (Parker, USA). The injection of EN (100μg/μl) or P53-MO (2-4ng/μl) was performed to rescue the phenotype in the mutants. The embryos were raised in E3 egg water (containing sea salts in distilled water), which was changed every 24 hours. All procedures were approved by the faculty Ethics Committee of Children's Hospital of Fudan University.

## Luciferase single-strand annealing recombination analysis

TALEN efficiency *in vitro* was evaluated by luciferase activity, which was recovered through DNA repair. Briefly, 10 ng of Renilla plasmid, 50 ng of luciferase single-strand annealing (SSA) reporter plasmid (including targeting sequence) and 100 ng of TALEN plasmids were cotransfected into Hek293T cells in 24-well plates by Lipofectamine TM 2000 (Invitrogen, USA), while only the first two kinds of plasmids transfected were used as negative controls. After 48h of transfection, cells were lysed by the Dual-Luciferase Assay System (Promega, USA). Then, the luciferase activity was measured with a SYNERGYMx Luminescence Microplate Reader (BioTek, USA).

## DNA extraction and PCR for sequencing

Genomic DNA was extracted from 5~6 embryos at 2 dpf, larva at 3 or 5 dpf and fin clips of adult zebrafish. The samples were incubated in 50 μl of 50 mM NaOH at 95°C for 20 min, and then 1M Tris-HCL (PH 8.0) was added to the mixture. Genomic DNA, which was used as a template for *eftud2* amplification, was obtained from the supernatant after centrifugation at 12,000 rpm for 5 min. The primers for TALENs efficacy testing were as follows: F: 5’-CATTCCGAATAGTTCCTC-3’, R: 5’-GGGTTAATGTAATAGGGTTA-3’. Additionally, 2 pairs of primers were designed to validate *eftud2* morpholinos, one of which was located in the exon region before and after the target site (EMO1: F-5’TCGCCATGGAGACTGATCTT3’, R-5’ CCACCTCAGGACCATACACT3’), and the sense primer of the other one was on the exon before the target site, while the anti-sense primer was in the intron region after the target area (EMO2: F-5’GAGCTAGATGCAGAGGACCG3’, R-5’ TGCGTTAGCCACTCAAAGGT3’). The PCR program was 95°C for 5 min, 35cycles of 95°C for 30 s, 56°C for 30 s, 72°C for 40 s, and a final extension at 72°C for 7min. After validation by agarose gel electrophoresis, the PCR product was sequenced directly or digested by T7E1 enzyme.

## Real-time PCR

Total RNA was extracted from cells or zebrafish samples using Trizol reagent (Ambion, USA), and cDNA was obtained from 1µg of total RNA by PrimeScript cDNA Synthesis Kit (Takara, Japan). The primers used for human and zebrafish are available in the supplementary materials (Table S2). The cycling profile was set at 95°C for 1 min, followed by 40 cycles of 95°C for 15 sec, 60°C for 30 sec, and 72°C for 30 sec. Comparative Ct values normalized to β-actin expression were determined for analysis.

## Western blotting

Cells and zebrafish samples were dissolved and homogenized in a mixture of RIPA lysis buffer, protease inhibitor (Thermo, USA) and phosphatase inhibitor (Roche, Swiss). Total protein was subjected to SDS-PAGE and transferred to nitrocellulose membranes, which were incubated with primary polyclonal antibodies against EFTUD2 (Rabbit, 1:5000, Abcam), phosphorylated P53 (Rabbit, 1:600, Abcam), and β-actin (Mouse, 1:2000, Sigma) or GAPDH (Mouse, 1:2000, Sigma) overnight at 4 ºC. Then, the membranes were incubated with second HRP-conjugated antibodies for 1 hour. The protein bands were visualized with the LAS400 imaging system (FUJI, Japan).

Table S1: Candidate genes with *de novo* mutations in the proband

| Gene.  Refgene | Ref | Alt | Func.  Refgene | GeneDetail.  Refgene |
| --- | --- | --- | --- | --- |
| AIM1 | CA | - | exonic | frameshift deletion |
| ANKLE1 | - | GT | exonic | stoploss |
| DMRTA2 | GCGGC | - | exonic | frameshift deletion |
| DNAH9 | CGGGCCGCGCTCGCGGC | - | exonic | frameshift deletion |
| EFTUD2 | CA | - | exonic | frameshift deletion |
| FGGY | CTGTATAT | - | exonic | frameshift deletion |
| KCNJ12 | AG | - | exonic | frameshift deletion |
| LTB4R2 | TGCACGGGGGCGACCGCTGGCGGCC | - | exonic | frameshift deletion |
| OR4C3 | TGCTGATC | - | exonic | frameshift deletion |
| PABPC1 | - | A | exonic | frameshift insertion |
| PSORS1C1 | C | - | exonic | frameshift deletion |
| UMODL1 | - | TCCA | exonic | frameshift insertion |
| ZBTB6 | ATAG | - | exonic | frameshift deletion |

Table S2: Real-time PCR primers of relative genes in cells

| Gene | Primer (5’－3’) | Length (bp) |
| --- | --- | --- |
| EFTUD2 | FP: GAGGTGGAGACCATAGTTCAAGA | 243 |
|  | RP: TTCCGGGTGAGTCTGTTCAAT |  |
| ALP | FP: ACCACCACGAGAGTGAACCA | 79 |
|  | RP: CGTTGTCTGAGTACCAGTCCC |  |
| COL1A1 | FP: GAGGGCCAAGACGAAGACATC | 140 |
|  | RP: CAGATCACGTCATCGCACAAC |  |
| OPN | FP: CTCCATTGACTCGAACGACTC | 230 |
|  | RP: CAGGTCTGCGAAACTTCTTAGAT |  |
| BMP2 | FP: ACCCGCTGTCTTCTAGCGT | 180 |
|  | RP: TTTCAGGCCGAACATGCTGAG |  |
| BMP4 | FP: AAAGTCGCCGAGATTCAGGG | 135 |
|  | RP: GACGGCACTCTTGCTAGGC |  |
| COLX | FP: ATGCTGCCACAAATACCCTTT | 107 |
|  | RP: GGTAGTGGGCCTTTTATGCCT |  |
| SOX9 | FP: AGCGAACGCACATCAAGAC | 85 |
|  | RP: CTGTAGGCGATCTGTTGGGG |  |
| FAS | FP: TCTGGTTCTTACGTCTGTTGC | 197 |
|  | RP: CTGTGCAGTCCCTAGCTTTCC |  |
| STEAP3 | FP: CTCCCCGGAGGTCATCTTTG | 117 |
|  | RP: TCTTGCTCTGTAGGGTTGCTC |  |
| CASP3 | FP: CATGGAAGCGAATCAATGGACT | 139 |
|  | RP: CTGTACCAGACCGAGATGTCA |  |
| P21 | FP: CCTGTCACTGTCTTGTACCCT | 130 |
|  | RP: GCGTTTGGAGTGGTAGAAATCT |  |
| SESN1 | FP: TGCTTTGGGCCGTTTGGATAA | 131 |
|  | RP: TGTAGTGACGATAATGTAGGGGT |  |
| β-actin | FP: CATGTACGTTGCTATCCAGGC | 250 |
|  | RP: CTCCTTAATGTCACGCACGAT |  |

**Fig. S1 *eftud2* expression pattern in WT zebrafish.** A: *eftud2* expression in zebrafish embryos at 24hpf (a, b), 48hpf (c, d), 3dpf (e, f) and 5dpf (g, h) was examined using whole-mount *in situ* hybridization over a period of 5 days, employing a specific *eftud2* anti-sense probe; (a, c, e, g) lateral view, (b, d, f, h) dorsal view. B: Relative mRNA levels of *eftud2* during the early developmental stages. C: Relative mRNA levels of *eftud2* in adult zebrafish tissues.

**Fig. S2 Targeting site and efficacy of *eftud2*-targeted TALEN mRNAs.** We also prepared three TALENs to construct the knockout zebrafish model, and the second one (T2) was the most effective for *in vitro* screening. A, B: The TALEN (T2) was designed at the first exon, which is presented in capital letters. C: *In vitro* efficacy was evaluated based on relative luciferase activity in TALEN-transfected Hek293T cells and the negative control. D: PCR products of *eftud2* containing the target sequence could be digested by the T7E1 enzyme, in which the product from the mutant zebrafish was cleaved into two fragments, whereas that from WT zebrafish was intact. E: Sequencing results of F0 generation showed mixed signals from the target site, which may predict the combination with TALEN mRNA and the *eftud2* gene.

**Fig. S3 Zebrafish with *eftud2* gene knockdown showed aberrant cartilage development.** A, B: Larvae treated with an *eftud2* morpholino (EMO) at 3dpf exhibited disrupted formation of Meckel’s cartilage (a) and the ceratohyals (b) upon alcian blue and alizarin red staining compared with the WT fish, fish injected with a mismatch morpholino (EMIS-MO) and fish rescued with normal human EFTUD2 mRNA (Rescue). A shows the lateral view, and B shows the ventral view. C, D: Bone and cartilage staining among different groups of larvae (WT, EMO, rescue, EMIS-MO) also suggested abnormal cartilage development at 5dpf.c, the ethmoid bones.

**Fig. S4 *EFTUD2* gene knockdown in HC-a.** A: Expression of *EFTUD2* mRNA in HC-a cells transfected with sh2 and sh3 lentivirus was lower than that in the shNT control.B: Protein expression of *EFTUD2* decreased in HC-a cells transfected with sh2 and sh3 lentivirus. C: Cell proliferation of HC-a cells transfected with sh2 and sh3 lentivirus was disrupted compared with that of the shNT control. D-E: *COL10A1* mRNA levels among different groups (shNT, sh2, sh3) of HC-a cells at 3 days before cell confluence, 3 days after cell confluence and 2 weeks after cell confluence. F-G: *SOX9* mRNA levels in HC-a cells among different groups (shNT, sh2, sh3) at 3 days before and after cell confluence. H: Alcian blue staining of HC-a cells among different groups (transfected with shNT, sh2 and sh3 lentiviruses). *: P˂0.05, **: P˂0.01, ***: P<0.001.

**Fig. S5 Differentially expressed genes identified through RNA-Seq analysis.** A: Correlation analysis between HCO cells transfected with shNT (Group1) or sh2 lentivirus (Group2), which showed a close relationship between the two groups. B: Genes of Group 1 and Group2 are located on all of the chromosomes. C: There were 6 genes involved in the P53 pathway, including *P21*, *FAS*, *STEAP3*, *CASP3*, *SESN1* and *THBS*. All of these genes showed elevated expression, except *THBS*, which was downregulated.
