# Supplementary figures and images for: EFTUD2 gene deficiency disrupts osteoblast maturation and inhibits chondrocyte differentiation via activation of the p53 signaling pathway
Source: Hum Genomics. 2019 Dec 5;13:63. doi: 10.1186/s40246-019-0238-y (PMC6894506; doi:10.1186/s40246-019-0238-y)

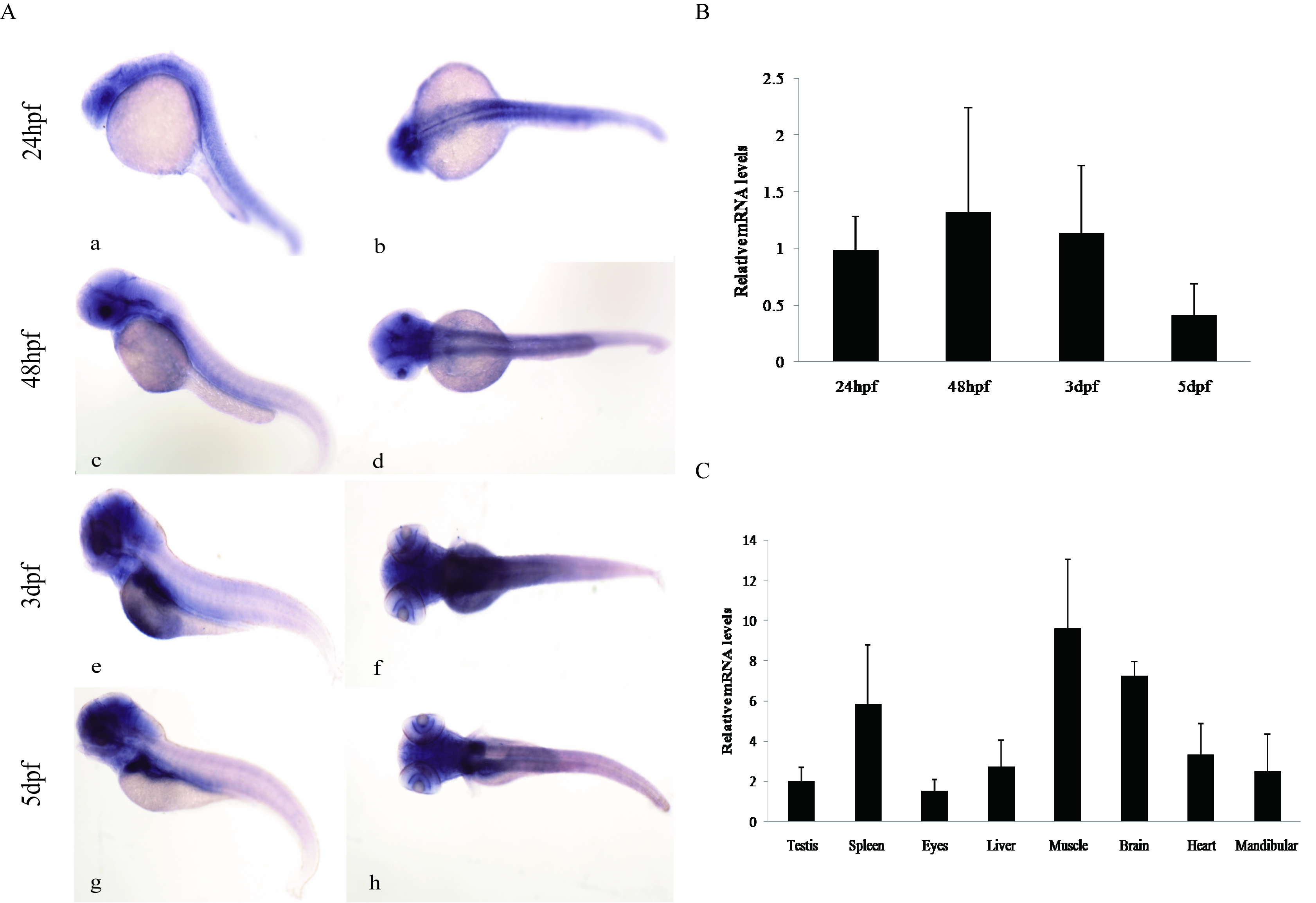

Supplement: Supplementary file 2 — Additional file 2: Figure S1. eftud2 expression pattern in WT zebrafish. A: eftud2 expression in zebrafish embryos at 24hpf (a, b), 48hpf (c, d), 3dpf (e, f) and 5dpf (g, h) was examined using whole-mount in situ hybridization over a period of 5 days, employing a specific eftud2 anti-sense probe; (a, c, e, g) lateral view, (b, d, f, h) dorsal view. B: Relative mRNA levels of eftud2 during the early developmental stages. C: Relative mRNA levels of eftud2 in adult zebrafish tissues. [file 40246_2019_238_MOESM2_ESM.tif]

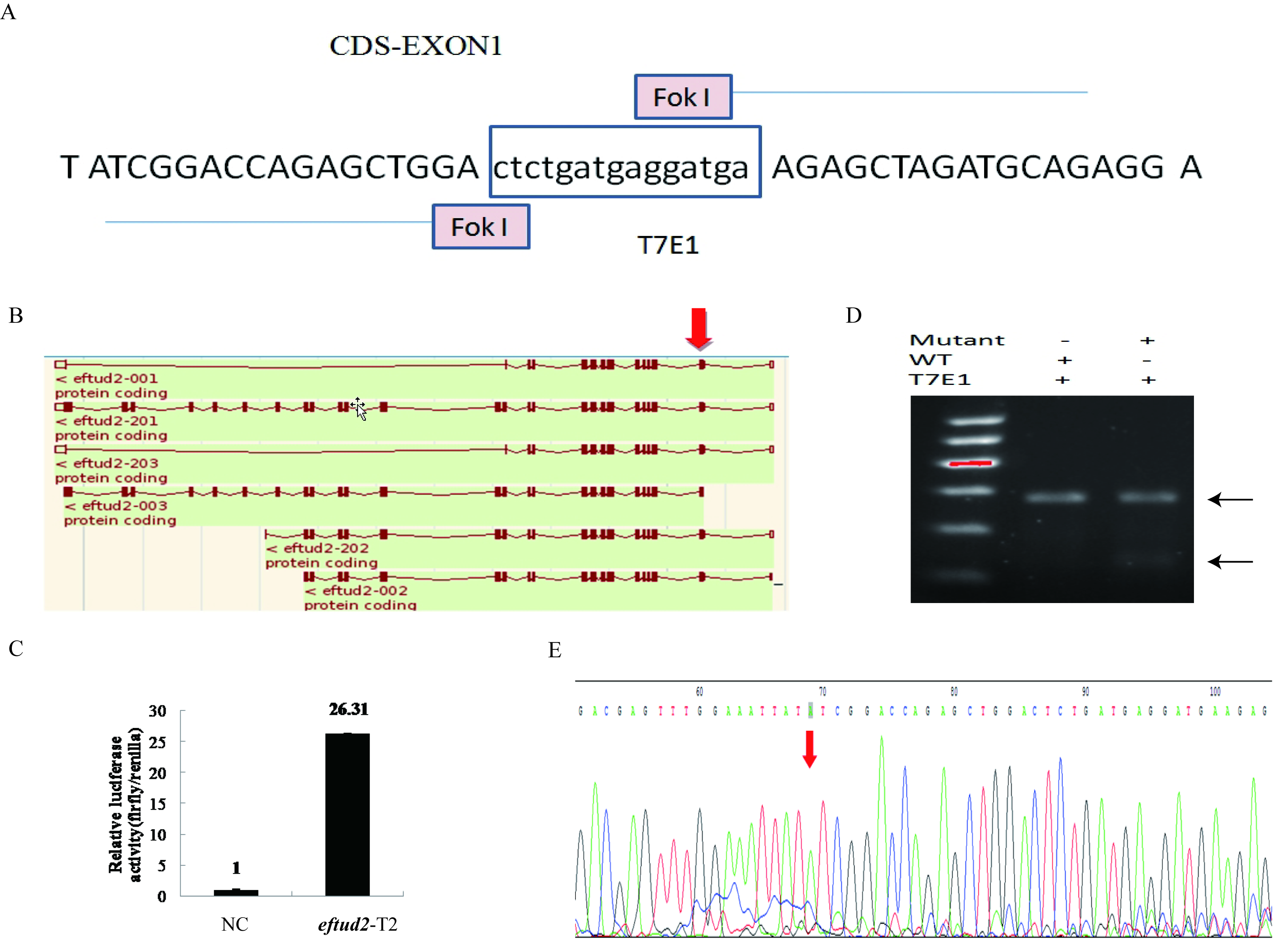

Supplement: Supplementary file 3 — Additional file 3: Figure S2. Targeting site and efficacy of eftud2-targeted TALEN mRNAs. We also prepared three TALENs to construct the knockout zebrafish model, and the second one (T2) was the most effective for in vitro screening. A, B: The TALEN (T2) was designed at the first exon, which is presented in capital letters. C: In vitro efficacy was evaluated based on relative luciferase activity in TALEN-transfected Hek293T cells and the negative control. D: PCR products of eftud2 containing the target sequence could be digested by the T7E1 enzyme, in which the product from the mutant zebrafish was cleaved into two fragments, whereas that from WT zebrafish was intact. E: Sequencing results of F0 generation showed mixed signals from the target site, which may predict the combination with TALEN mRNA and the eftud2 gene. [file 40246_2019_238_MOESM3_ESM.tif]

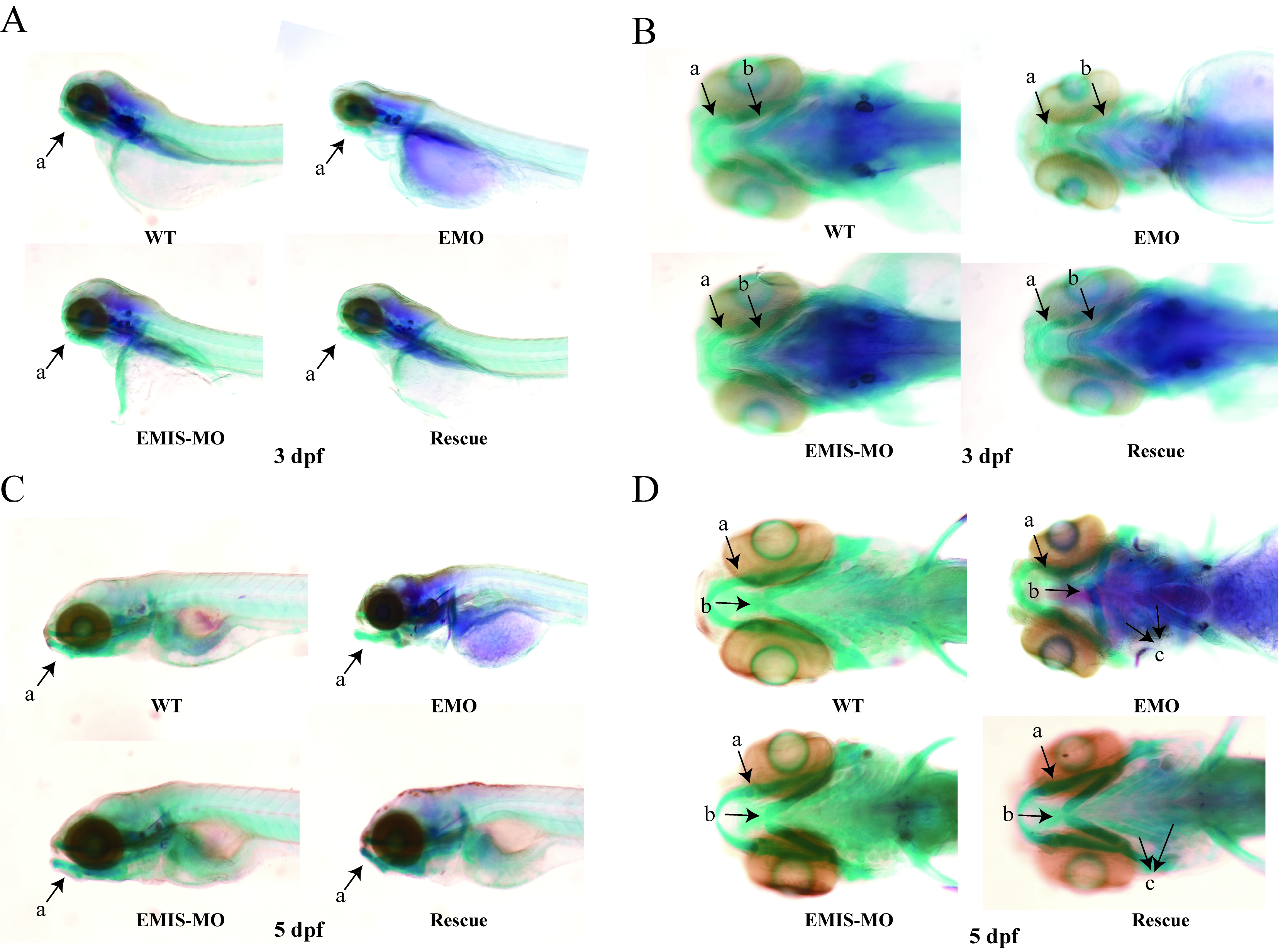

Supplement: Supplementary file 4 — Additional file 4: Figure S3. Zebrafish with eftud2 gene knockdown showed aberrant cartilage development. A, B: Larvae treated with an eftud2 morpholino (EMO) at 3dpf exhibited disrupted formation of Meckel’s cartilage (a) and the ceratohyals (b) upon alcian blue and alizarin red staining compared with the WT fish, fish injected with a mismatch morpholino (EMIS-MO) and fish rescued with normal human EFTUD2 mRNA (Rescue). A shows the lateral view, and B shows the ventral view. C, D: Bone and cartilage staining among different groups of larvae (WT, EMO, rescue, EMIS-MO) also suggested abnormal cartilage development at 5dpf.c, the ethmoid bones. [file 40246_2019_238_MOESM4_ESM.tif]

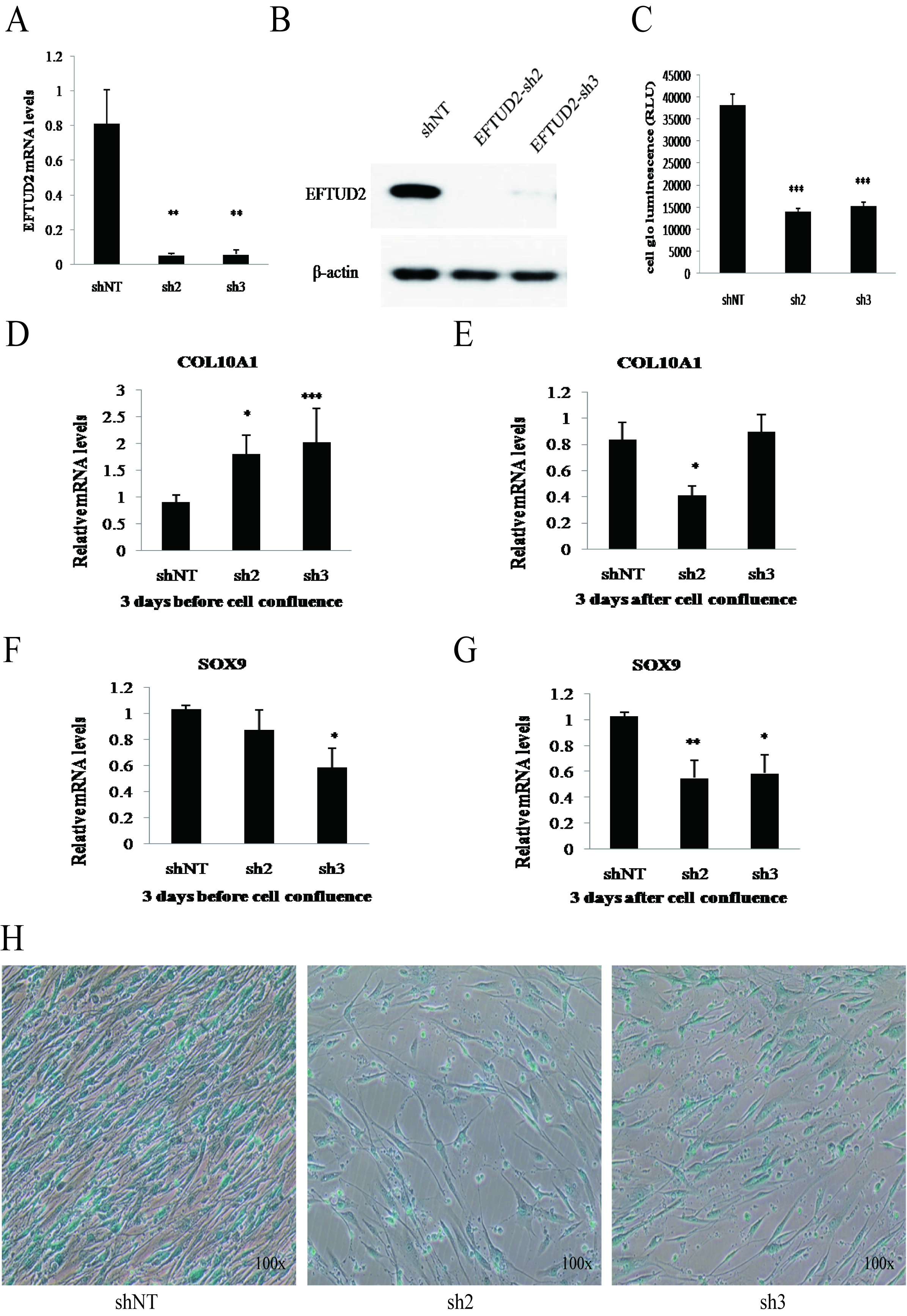

Supplement: Supplementary file 5 — Additional file 5: Figure S4. EFTUD2 gene knockdown in HC-a. A: Expression of EFTUD2 mRNA in HC-a cells transfected with sh2 and sh3 lentivirus was lower than that in the shNT control.B: Protein expression of EFTUD2 decreased in HC-a cells transfected with sh2 and sh3 lentivirus. C: Cell proliferation of HC-a cells transfected with sh2 and sh3 lentivirus was disrupted compared with that of the shNT control. D-E: COL10A1 mRNA levels among different groups (shNT, sh2, sh3) of HC-a cells at 3 days before cell confluence, 3 days after cell confluence and 2 weeks after cell confluence. F-G: SOX9 mRNA levels in HC-a cells among different groups (shNT, sh2, sh3) at 3 days before and after cell confluence. H: Alcian blue staining of HC-a cells among different groups (transfected with shNT, sh2 and sh3 lentiviruses). *: P < 0.05, **: P < 0.01, ***: P < 0.001. [file 40246_2019_238_MOESM5_ESM.tif]

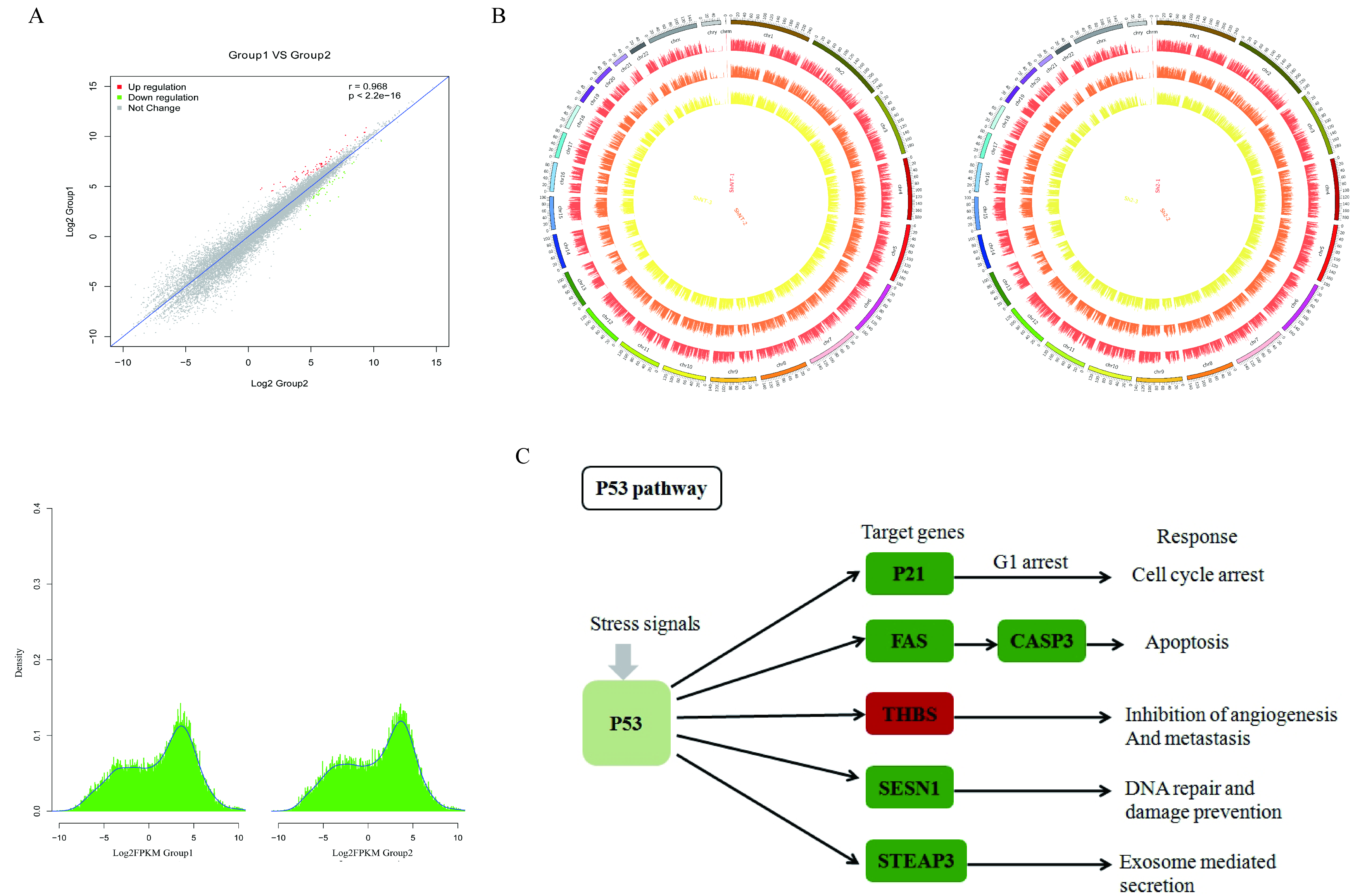

Supplement: Supplementary file 6 — Additional file 6: Figure S5. Differentially expressed genes identified through RNA-Seq analysis. A: Correlation analysis between HCO cells transfected with shNT (Group1) or sh2 lentivirus (Group2), which showed a close relationship between the two groups. B: Genes of Group 1 and Group2 are located on all of the chromosomes. C: There were 6 genes involved in the P53 pathway, including P21, FAS, STEAP3, CASP3, SESN1 and THBS. All of these genes showed elevated expression, except THBS, which was downregulated. [file 40246_2019_238_MOESM6_ESM.tif]
